# Mutational Analysis of Intracellular Loops Identify Cross Talk with Nucleotide Binding Domains of Yeast ABC Transporter Cdr1p

**DOI:** 10.1038/srep11211

**Published:** 2015-06-08

**Authors:** Abdul Haseeb Shah, Manpreet Kaur Rawal, Sanjiveeni Dhamgaye, Sneha Sudha Komath, Ajay Kumar Saxena, Rajendra Prasad

**Affiliations:** 1 School of Life Sciences, Jawaharlal Nehru University, New Delhi 110067, India

## Abstract

The ABC transporter Cdr1 protein (Cdr1p) of *Candida albicans,* which plays a major role in antifungal resistance, has two transmembrane domains (TMDs) and two nucleotide binding domains (NBDs) that are interconnected by extracellular (ECLs) and intracellular (ICLs) loops. To examine the communication interface between the NBDs and ICLs of Cdr1p, we subjected all four ICLs to alanine scanning mutagenesis, replacing each of the 85 residues with an alanine. The resulting ICL mutant library was analyzed by biochemical and phenotypic mapping. Only 18% of the mutants from this library displayed enhanced drug susceptibility. Most of the drug-susceptible mutants displayed uncoupling between ATP hydrolysis and drug transport. The two drug-susceptible ICL1 mutants (I574A and S593A) that lay within or close to the predicted coupling helix yielded two chromosomal suppressor mutations that fall near the Q-loop of NBD2 (R935) and in the Walker A motif (G190) of NBD1. Based on a 3D homology model and kinetic analysis of drug transport, our data suggest that large distances between ICL residues and their respective chromosomal suppressor mutations rule out a direct interaction between them. However, they impact the transport cycle by restoring the coupling interface via indirect downstream signaling.

The incidence of opportunistic fungal infections has increased over the past few decades because of comorbidity with other infectious diseases such as tuberculosis, malaria and AIDS as well as due to medical interventions that render patients immunocompromised^1^,^2^. Infections caused by *Candida albicans* have also increased in frequency in the past three decades, primarily due to AIDS and modern medical practices^1^. Infections caused by *C. albicans* are commonly treated with either azoles or non-azole antifungal drugs^2^,^3^. Widespread and prolonged use of azoles in recent years has led to increased drug tolerance, leading to persistent infections. Over the years, several mechanisms that contribute to the development of multidrug resistance (MDR) in pathogenic fungi including the human fungal pathogen *C. albicans* have been identified^4^. Various mechanisms contribute to this resistance, including overexpression of genes that encode drug efflux pump proteins of two transporter superfamilies. These include the ATP-binding cassette (ABC) transporters, encoded by *CDR1* and *CDR2*^5^,^6^ and the major facilitator superfamily (MFS) transporter encoded by *MDR1*^7^,^8^. Various studies have identified *Candida* drug resistance protein 1 (Cdr1p) as the major drug efflux pump involved in the development of resistance to azole antifungals. Notably, Cdr1p overexpression also coincides with an increased drug substrate efflux in azole resistant (AR) clinical isolates^3^,^9^.

Cdr1p, the 1501 amino acid long integral plasma membrane (PM) transporter of *C. albicans*, has structural features common to other full ABC transporters, including two cytoplasmic nucleotide binding domains (NBDs) and two transmembrane domains (TMDs)^5^,^10^. Unlike the forward (TMD-NBD)_2_ topology found in mammalian MDR1/P-glycoprotein (ABCB1, P-gp), the fungal pleiotropic drug resistance (PDR) ABCG transporters such as Cdr1p are predicted to have a reverse (NBD-TMD)_2_ topology^11^,^12^. Each of the two TMDs of Cdr1p is composed of six helical transmembrane segments (TMSs) that apparently harbor drug binding sites and thus impart substrate specificity to the protein^10^,^13^. In response to drug binding in the TMDs, the NBDs bind ATP. This enables a conformational change that results in drug translocation followed by conformational resetting due to ATP hydrolysis by the NBDs. This cyclic process is thought to involve a series of interfacial contacts between TMDs, between the TMDs and NBDs and between the NBDs^11^,^12^.

Of note, the 12 TMSs of yeast ABC transporters are interlinked by six extracellular loops (ECL1-6) on the extracellular face and four intracellular loops (ICL1-4) on the cytosolic face of the PM. ECLs are less conserved at the sequence level and have comparable length to other transporters, while ICLs have mostly conserved but shorter primary sequences^10^,^14^. Functional studies so far suggest that ECLs contribute to drug specificity and affect the transport of drug substrates, while ICLs generally serve as intra-domain communication interface between TMDs and NBDs^10^,^14^. However, the exact role of ECLs and ICLs in drug transport is not well understood.

Crystal structures of non-yeast ABC transporters suggest that both TMDs contact the NBDs at a communication interface provided by the ICLs^15^,^16^,^17^. Biochemical studies of the *Saccharomyces cerevisiae* ABCG transporter Pdr5p have provided evidence that ICLs link the TMDs and NBDs^18^,^19^,^20^ and that the ICLs affect protein folding and membrane localization in Yor1p, the yeast homologue of human cystic fibrosis transmembrane conductance regulator CFTR^21^,^22^. Despite evidence supporting inter domain communication, demonstrating the importance of such interactions for yeast transporters is a challenge. Considering the contribution of Cdr1p to triazole resistance, greater mechanistic understanding of the role of the ICLs could assist the design of inhibitor/modulators of this xenobiotic transporter. In the absence of yeast ABC transporter crystal structures, we have used bioinformatics tools to identify the four Cdr1p ICLs and subjected them to scanning alanine mutagenesis in a yeast host that heterologously over expresses Cdr1p. Phenotypic and biochemical analysis showed that only 12 out of 85 mutants showed enhanced drug susceptibility to two or more drugs. Further analysis of these mutants, including two suppressor mutants plus site directed mutations in a Walker A motif and in a Q-loop are consistent with their role in communication between NBD and TMDs mediated by Cdr1p ICLs.

## Results

### Cdr1p ICLs show length and sequence conservation

**Figure 1:**
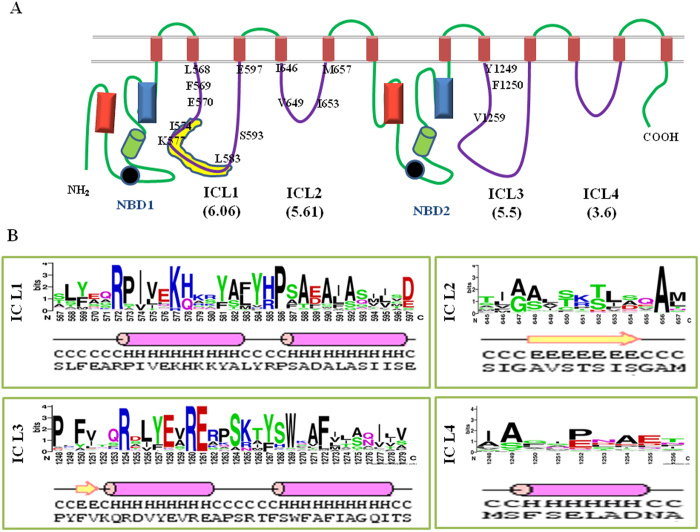
Schematic representation of Cdr1p showing intracellular loops and their structural features. **A**) Four intracellular loops of Cdr1p showing the numbered residues that cause drug-susceptible phenotype when replaced with alanine. The values in parentheses below each ICL show loop conservation scores (LCS) calculated as the ratio between sum of conservation scores of all residues positions in each loop by the total no of residues in that loop. The values are arbitrary based on the conservation of individual amino acids of Cdr1p on alignment with 85 fungal ABCG transporters^23^. The conservation scores were obtained using JALVIEW2.4.0.b2 ( http://www.jalview.org). Different conserved structural elements in each NBD are indicated by different colored shapes, red colored rectangles for Walker A, blue colored rectangles as Walker B, Signature motif as green colored cylinders and Q-loops as black circles. Yellow patch in ICL1 depicts predicted coupling helix from position 573 to 582. **B**) Structural features of Cdr1p ICL residues that show conservation of residues among 85 fungal ABCG transporters determined from their aligned sequences by WebLogo ( http://weblogo.berkeley.edu/logo.cgi). The bottom row of the panel depicts predicted secondary structural arrangements of ICL residues.

In this study, we have evaluated the role of the Cdr1p ICLs, which interconnect the twelve TMSs within the two TMDs. Initially, a multiple sequence alignment (MSA) of 85 full-length fungal ABCG transporters that have topology similar to Cdr1p^23^ was used to delineate and to accurately determine the length and placement of Cdr1p ICLs for comparison with other ABCG transporters. Our analysis suggests that the four Cdr1p ICLs are distributed equally among the two TMDs. Thus, ICL1 and ICL2 for TMD1 and ICL3 and ICL4 for TMD2 act as interconnecting cytoplasmic loops. Similar to other members of the ABCG transporter family, ICL1 and ICL3 from Cdr1p are comparatively longer, consisting of 31 and 32 residues, respectively, than ICL2 and ICL4 which contain 13 and 9 residues, respectively (Fig. 1A). Secondary structural arrangement show that ICL1 and ICL3 are quite similar, with each loop predicted to contain two helix forming stretches, while ICL2 and ICL4 are predicted to contain a beta sheet and a helix structure, respectively (Fig. 1B). The conservation of loops was compared by calculating loop conservation scores (LCS) which were calculated as the ratio of sum of conservation scores of all residue positions in each loop by the total number of residues in that loop. For example, for ICL1 the conservation scores for 31 amino acids was added up to give a total conservation score of 188 which, when divided by the total number of residues in ICL1, gives a conservation score of 6.06 (Supplementary Table S1)^23^. The average conservation scores of these ICLs decrease across the protein sequence; ICL1 is the most conserved, and ICL4 is the least conserved (Fig. 1A).

Cdr1p ICL1 contains a centrally located coupling helix (CH) similar to the structure predicted for other ABCG transporters^14^. In Cdr1p, the ICL1 CH (^573^PIVEKHKKYA^582^) is ten amino acids long and is highly conserved with an average conservation score of 6.1 and includes two highly conserved residues, I574 and K577. The coupling helix in ICL1 is shown by yellow colored patch in Fig 1A. ICL1 contains negatively charged amino acids at each end, connecting ICL1 with its corresponding TMS (Fig 1A). To gain insight into the importance of these cytoplasmic loops, we have subjected the entire primary structure of all the ICLs to site directed mutagenesis, replacing each of the 85 ICL residues individually with alanine. The mutants were confirmed before being subjected to phenotypic and biochemical analysis.

### Replacement of conserved ICL residues predominantly yields neutral mutations

**Figure 2:**
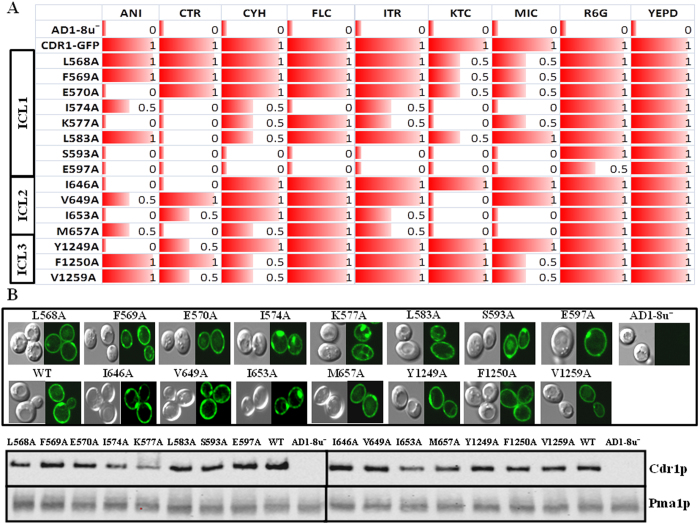
Drug susceptibility, membrane localization and expression of Cdr1p ICL mutant variants. **A**) Represents the drug susceptibility profile in the presence of different drugs as observed in spot assays. Values 0, 0.5 and 1 or corresponding red bars, respectively represent no growth, partial growth and full growth on indicated drug at given concentrations. Drug concentrations used are ANI, 2 μg/ml, CTR 0.4 μg/ml, CYH 0.05 μg/ml, FLC 3 μg/ml, ITR 2 μg/ml, KTC 0.08 μg/ml, MIC 0.08 μg/ml, R6G 3 μg/ml. ***B**,* Membrane localization of drug-susceptible Cdr1p-GFP ICL mutants observed by confocal microscopy (above) and western blots (below) show the expression of Cdr1p-GFP probed with an anti-GFP monoclonal antibody. The membrane protein Pma1p was probed with an anti-Pma1 antibody as a loading control and indicator of PM localization.

Alanine scanning of the ICL residues revealed that the replacement of most of the 85 total amino acid residues by an alanine resulted in a neutral phenotype in the presence of various drugs, comparable to that of cells expressing WT Cdr1p. Out of the 12 residues that are highly conserved in the Cdr1p ICLs, only 2 residues (I574 and K577) showed enhanced drug-susceptibility upon replacement with alanine. A majority of the drug-susceptible ICL mutants resulted from replacement of non-conserved residues. Drug susceptibility profiling performed by two independent assays revealed that a total of 15 ICL residues became susceptible to two or more drugs upon replacement with alanine; however, their susceptibility pattern was variable. For example, among the various ICL mutants, 11 (L568A, F569A, E570A, I574A, K577A, S593A, E597A, I653A, M657A, F1250A, and V1259A) were susceptible to an imidazole drug, miconazole (MIC), but their susceptibility varied when tested with other imidazole drugs. Additionally, 8 ICL residues (I574A, K577A, L583A, S593A, E597A, I646A, Y1249A and V1259A) resulted in strains susceptible to clotrimazole (CTR) when replaced with alanine, while 11 variants (L568A, F569A, E570A, I574A, K577A, L583, S593A, E597A V649, I653A and M657A) generated strains that were susceptible to ketoconazole (KTC). Only 3 of the ICL mutants were susceptible to the triazole antifungal, fluconazole (FLC), and 6 were susceptible to itraconazole (ITR). All the mutants that were susceptible to triazoles were also simultaneously susceptible to imidazoles, while the reverse was not true. Susceptibility to both cycloheximide (CYH) and anisomycin (ANI) was observed in 8 (I574, K577A, L583A, S593A, E597A, M657A, F1250A andV1259A) and 9 (E570A, I574A, S593A, E597, I646A, V649A, I653A, M657A and Y1249A) strains expressing the mutant variants, respectively. Figure 2A depicts the drug response profile by spot assays of those mutants, which displayed increased susceptibility to two or more drugs. Most of the MIC_80_ values obtained by the broth micro dilution method given in Table 1, corroborated with the results obtained with the spot assays, however, there were few mutants where two assays data do not strictly match. Such inconsistency between the assays has been observed earlier as well^24^. The rest of the mutants showing phenotype comparable to WT or showing susceptibility to a single drug are shown in Supplementary Fig. S1 A–D and were not analyzed further.

### Cdr1p ICL mutant variants are not misfolded

ICL mutations in close Cdr1p homologs such as *S. cerevisiae* Yor1p, human P-glycoprotein (ABCB1), multidrug resistance protein 1 (MRP1) and in cystic fibrosis transmembrane conductance regulator (CFTR), have been observed to cause misfolding and maturation defects^21^,^25^,^26^,^27^. We examined the localization of GFP-tagged Cdr1p ICL mutant variants. As shown in confocal images in Fig. 2B (upper panel), most ICL variants appeared to be correctly PM localized. They showed fluorescence at the cell surface typical of GFP-tagged Cdr1p with the exception of the K577A variant, which appears to be partially trapped within intracellular structures. The membrane localization was further evaluated by western blot analysis which confirmed that all the drug-susceptible mutants were properly expressed and localized to the PM at levels comparable to WT protein with minor variations in some cases (Fig. 2B, lower panel). Together, these results excluded the possibility that the observed Cdr1p ICL mutant phenotypes could be due to mislocalization or poor expression of the protein variants.

We also explored whether the decreased drug susceptibility of the ICL mutants was a result of non-native protein conformation^22^,^28^. Thus, PM fractions isolated from cells expressing either native or mutant variant proteins were subjected to limited trypsin digestion as described in the methods. Only six ICL mutants with increased susceptibility to maximum number of tested drugs (four or more) were subjected to trypsin digestion. As shown in Supplementary Fig. S2, native and ICL mutant proteins possess a number of exposed trypsin sites that could result in several digestion products. The main digestion products obtained were around 100, 50 and 25 kD size in limited proteolysis. The pattern of digestion of the mutant variant proteins closely resembled that of the native protein, thus implying that gross conformational changes associated with ICL mutations are unlikely.

### Most ICL mutants display normal ATPase and drug efflux activities

**Figure 3:**
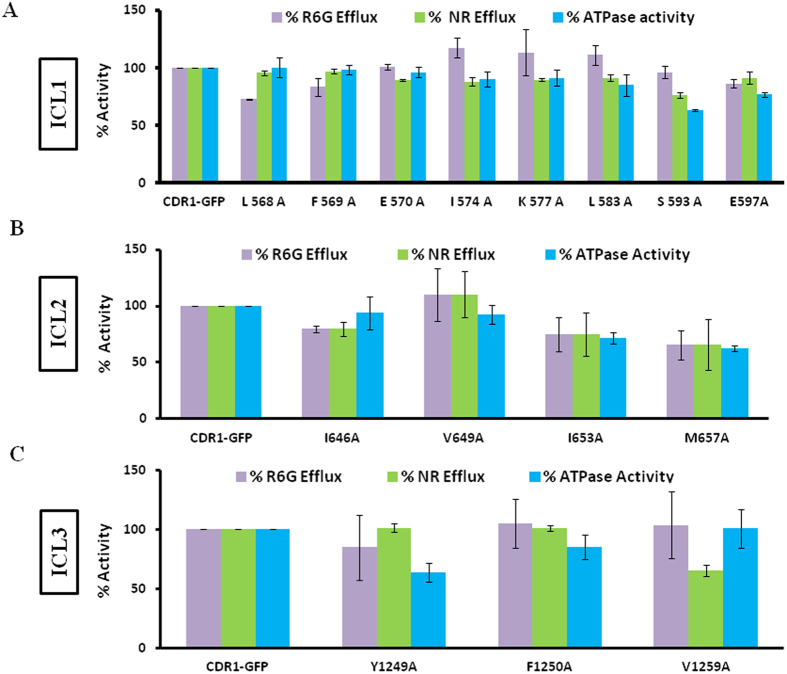
Substrate transport and ATPase activity of drug-susceptible Cdr1p ICL1, ICL2, and ICL3 mutants. Percentage efflux of R6G and NR was measured and compared to its efflux in WT Cdr1p, which was set at 100%. OM-sensitive ATPase activity of the Cdr1p mutants was performed using 10 μg protein with incubation at 30 °C for 30 min and was compared to that of WT Cdr1p, which was considered 100% activity. The released inorganic phosphate was detected colorimetrically at 880 nm as described in the methods. **A**,**B**,**C**) represents the activities in susceptible mutants from ICL1, ICL2 and ICL3, respectively.

Cdr1p efficiently transports fluorescent substrates such as rhodamine 6G (R6G) and nile red (NR) that are commonly used as an index of protein transport properties. In the present case, none of the ICL mutants were susceptible to the tested fluorescent substrates. Expectedly, therefore, efflux of the fluorescent substrates such as of R6G and NR in ICL mutants also remained largely unaffected (Fig. 3A–C). However for a few mutant variants such as L568A, I646A and V1259A, the efflux was reduced by upto 30%. The ICL mutants I574A, S593A and E597A were, also selectively susceptible to FLC. We measured the accumulation of radiolabelled FLC (^3^H-FLC) in these mutants as described in the methods. The accumulation of ^3^H-FLC was substantially higher in I574A, S593A and E597A mutants (discussed below), which agrees with their enhanced susceptibility to this triazole (Fig. 2A). Whether the other drug-susceptible ICL mutants also show decreased efflux of the drugs for which they were susceptible could not be ascertained due to the lack of suitable substrates to measure their transport. However, considering the toxicity of certain xenobiotics towards the cells expressing ICL mutant variants as compared to the host strain (Fig. 2A), the efflux of the tested toxic substrates is also expected to be defective in these mutants.

Similar to other yeast homologues, Cdr1p also elicits high basal ATPase activity. However, unlike mammalian homologues, yeast ABC transporters including Cdr1p do not display drug-stimulated ATPase activity. Furthermore, while drug binding to Cdr1p is ATP independent, ATP hydrolysis is coupled to drug extrusion^10^. We examined whether ATPase activity of Cdr1p is affected by the ICL mutations. Most of the ICL mutants retained near normal ATPase activity that was comparable to that of the native protein (Fig. 3A–C). Considering the drug susceptibility data, ATP hydrolysis seems to be uncoupled from substrate transport, thereby affecting the transport of selected drugs.

### Drug susceptibilities of ICL mutants are significantly reduced by suppressor mutation in the Walker A motif of NBD1 or near the Q loop region of NBD2

**Figure 4:**
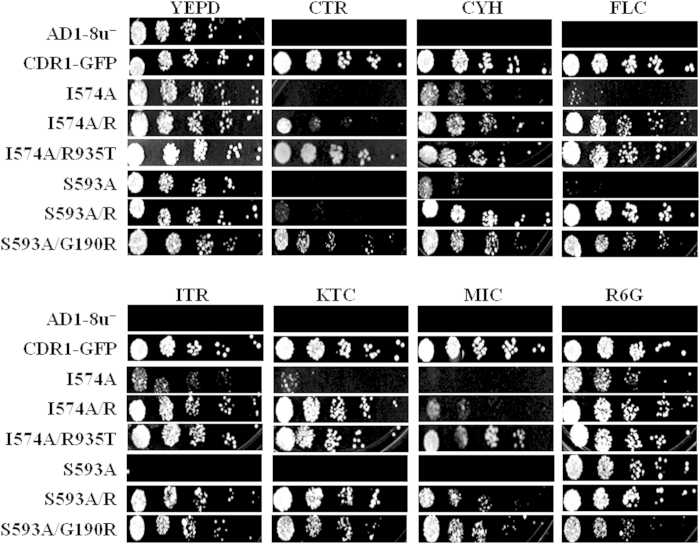
Drug resistance profile of two ICL mutants, I574A and S593A, and their chromosomal suppressor mutants. Spot assay shows the drug susceptibility profile of ICL1 mutants and their natural chromosomal suppressors and corresponding suppressors generated in respective plasmids by site directed mutagenesis. R-denotes natural chromosomal suppressor mutants. Drug concentrations used are, ANI 2 μg/ml, CTR 0.4 μg/ml, CYH 0.05 μg/ml, FLC 3 μg/ml, ITR 2 μg/ml, KTC 0.08 μg/ml, MIC 0.08 μg/ml, R6G 3 μg/ml.

To gain mechanistic insight into the hyper susceptibility of ICL mutants, we looked for spontaneous yeast suppressor mutants that could complement their functional deficiencies. Our strategy included plating each selected single drug-susceptible ICL mutant on a plate containing a toxic concentration of the indicated drug (see methods). Suppressor isolates within the inhibition zones appeared at a low frequency consistent with spontaneous mutation and stably reverted susceptibility to drug in drug susceptibility spot assays (Fig. 4) as well as in checkerboard liquid susceptibility assays (Supplementary Table S2). The drug resistance phenotype was maintained in all isolates after several passages on non-selective YEPD agar plates as well as on drug plates. ICL mutant strains that were susceptible to most of the drugs (I574A, K577A, S593A and E597A from ICL1; I653A and M657A from ICL2) were subjected to a suppressor screen; however, initially in our screen, only two ICL mutants I574A and S593A resulted in drug resistant colonies within drug inhibitory zones on agar plates. The chromosomal suppressor mutants generated against I574A were recovered in the presence of KTC and FLC, while S593A yielded suppressor colonies in the presence of KTC and CYH. Although, we recovered chromosomal repressors against the two ICL mutations grown in the presence of specific drugs, all the generated suppressors displayed collateral resistance to other drugs (Fig. 4). A maximum of three suppressor colonies obtained in the presence of each drug against individual ICL mutants were sequenced and analyzed as described in the methods. The sequencing of *CDR1* revealed that in case of I574A, all three suppressor colonies obtained in presence of FLC reverted to WT (A574I), while all three suppressor colonies obtained in presence of KTC mapped to R935, where the native arginine was replaced with threonine (R935T). The S593A suppressor colonies, which grew on CYH, did not have any sequence changes in the *CDR1* orf, thus ruling out any direct role of the *CDR1* gene in the observed reversal in phenotype. However, a complete nucleotide sequencing of the *CDR1* gene indicated that the suppressor colonies recovered against KTC mapped to a change at G190 in the Walker A motif of NBD1 where glycine was replaced with an arginine (G190R). Each chromosomal suppressor phenotype was further confirmed by recreating the suppressor mutations in pPSCDR1-GFP plasmid containing the *CDR1* gene by site directed mutagenesis (Fig. 4). Notably, while double mutant plasmids were phenotypically comparable to chromosomal revertant strains, the single suppressor mutation (either G190R or R935T) introduced by site directed mutagenesis resulted in WT phenotypes in presence of different drugs as shown in Fig. 4. It should be noted that in some instances the drug susceptibility pattern was not strictly comparable between naturally obtained chromosomal revertant strains and the corresponding suppressor mutants created by site directed mutagenesis. This reflects that chromosomal suppressors may also harbor mutation(s) elsewhere in genome as was the case with S593A chromosomal revertant strain obtained in presence of CYH (discussed above).

### Altered drug transport kinetics and ATPase activities of ICL mutants I574A and S593A are alleviated by suppressor mutations

**Figure 5:**
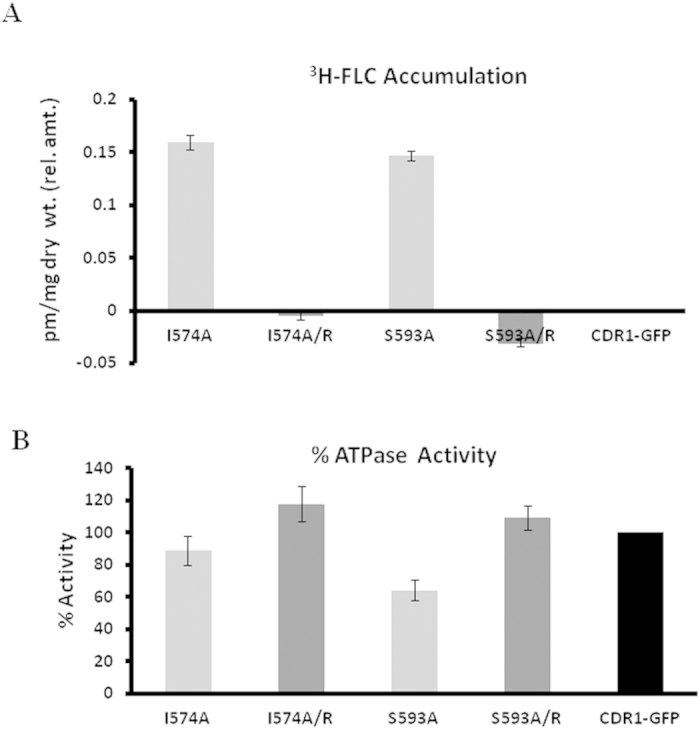
Relative ^3^H-FLC accumulation and ATPase activity of two ICL mutants, I574A and S593A, and their chromosomal suppressors. **A**) Accumulation of radiolabelled FLC inside drug-susceptible ICL1 mutants and their respective suppressor strains relative to the accumulation in WT Cdr1p strain. **B**) Percentage ATPase activity of ICL1 mutants and their respective suppressor strains compared with ATPase activity in WT Cdr1p taken as 100%.

Because I574A and S593A are also susceptible to FLC, we were able to analyze the transport and ATP hydrolysis capabilities of I574A and S593A strains in a well-established, whole cell based ^3^H-FLC accumulation assay as described in the methods. We compared the ^3^H-FLC accumulation capability of WT Cdr1p with I574A and S593A mutant strains. The data depicted in Fig. 5A show that both the I574A and S593A strains, which displayed increased susceptibility to FLC, also showed enhanced accumulation of ^3^H-FLC, suggesting reduced drug efflux. Between the two susceptible strains, S593A also showed a 40% reduction in ATPase activity while no major difference in ATPase activity was observed in I574A when compared to the WT strain (Fig. 5B). The observed abrogation of ^3^H-FLC transport and ATPase activity of ICL mutants were reversed in their respective suppressor strains. For example, the two chromosomal suppressors R935T and G190R recovered against I574A and S593A, respectively, showed decreased ^3^H-FLC accumulation compared to that of their corresponding drug-susceptible mutant strains (Fig. 5A). The reduced ATPase activity of the S593A mutant was also considerably recovered in the G190R suppressor strain (Fig. 5B)

A preliminary understanding of the mechanistic effects of the mutations was obtained by studying ATPase kinetics and ^3^H-FLC accumulation in the I574A and S593A ICL variants and in their respective chromosomal suppressors (Table 2). The decrease in ATPase activity along with an increase in drug susceptibility of S593A could partially be attributed to the approximately 1.6-fold lower V_max_ values (Table 2A). The decrease in V_max_ could most likely be attributed to poor ATP hydrolysis and/or greater affinity of the active site for the product (or slower kinetics for the product leaving the active site). The chromosomal suppressor mutation G190R recovered from the S593A variant showed partial reversal of kinetic parameters of ATPase activity compared to wild-type Cdr1p (Table 2A). The ICL mutant I574A did not show any substantial variation in ATPase activity, which was also reflected in its V_max_ or K_m_ values. The other kinetics parameters of ATP hydrolysis, such as k_cat_ and k_cat_/K_m_, exhibited no notable differences for I574A while k_cat_/K_m_ ratio showed increase in S593A mutant (Table 2A).

Because FLC drug resistance and transport decreased in both mutant strains (Fig. 5A) we measured the kinetics of ^3^H-FLC accumulations in mutant as well as in their suppressor strains to gain better insight into this process. Because we monitored ^3^H-FLC accumulation rather than efflux, we designated this quantity as A_max,_ denoting maximum accumulation. The results are depicted in Table 2B. Both mutant strains, I574A and S593A, displayed increased A_max_, which could contribute to enhanced susceptibility of these ICL mutants towards FLC. Interestingly, the A_max_ of FLC in both the alanine mutants was reverted back to WT strain levels in the suppressor strains (R935T and G190R). The K_d_ of FLC accumulation for both ICL mutants was also brought back to WT levels in their respective chromosomal suppressors.

### Suppressor mutant guided screen confirms the sequences around Q-loop and Walker A motifs important for maintaining inter domain communication

**Figure 6:**
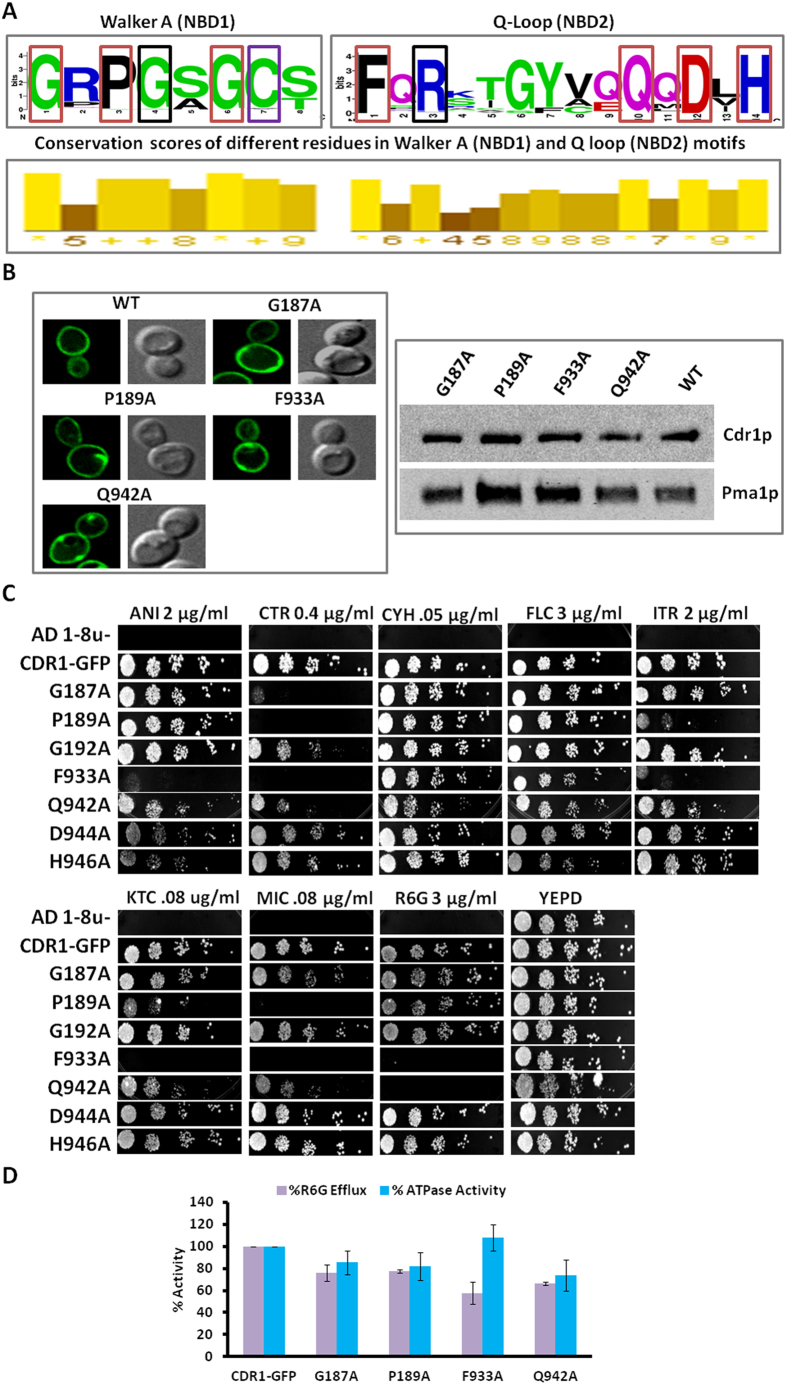
Characterization of mutants flanking suppressor mutants. **A**) Weblogo depicting conservation (upper panel) and conservation scores (lower panel) of the residues flanking suppressor mutations in NBD1 Walker A motif and NBD2 Q-Loop region. 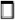 Natural chromosomal suppressor mutations obtained. 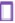 Mutation performed in earlier studies^29^,^30^. 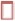 Mutations performed in Walker A of NBD1 or around Q-Loop of NBD2 having conservation scores similar or higher than suppressor mutants (G190R and R935T) recovered from corresponding motifs. **B**) Membrane localization of drug-susceptible Cdr1p-GFP NBD mutants observed by confocal microscopy, and western blot, show the expression of Cdr1p-GFP probed with an anti-GFP monoclonal antibody. The membrane protein Pma1p was probed with an anti-Pma1 antibody as a loading control and indicator of PM localization. **C**) Spot assay shows the drug susceptibility profile of NBD mutants flanking the suppressor mutants. Drug concentrations used are indicated in respective panels. **D**) Percentage efflux of R6G was measured and compared to its efflux in WT Cdr1p, which was set at 100%. OM-sensitive ATPase activity of the Cdr1p mutants was performed using 10 μg protein with incubation at 30 °C for 30 min and was compared to that of WT Cdr1p, taken as 100% activity. The released inorganic phosphate was detected colorimetrically at 880 nm as described in the methods.

To get further insight into the role of the residues, we made another round of mutations. Our rationale was based on the positioning of the already recovered suppressor mutations in NBD1 and NBD2 sub-domains. As depicted in the web-logo in Fig. 6A, all the conserved residues flanking suppressor G190 from Walker A of NBD1 and to R935 near Q-loop of NBD2 were subjected to site directed mutagenesis. Each of the amino acid residues in Walker A of NBD1 or around Q-loop of NBD2 having conservation scores higher or equal to the natural suppressor mutations obtained in this region were replaced individually with alanine (Fig 6A). It should be mentioned that the well conserved C193 was not included in this series of mutations since we have previously established its role in the ATP catalytic cycle^29^,^30^.

Thus, three amino acid residues from Walker A motif of NBD1 (G187, P189, G192) and four amino acid residues around Q-loop of NBD2 (F933, Q942, D944 and H946) were individually mutated to alanine. After confirming that all the new series of mutants of NBD1 (G187A, P189A, G192A) and NBD2 (F933, Q942A, D944A and H946A) were properly membrane localized and expressed, they were subjected to phenotypic analysis. The membrane localization of only drug susceptible mutants (discussed below) from this set is shown by confocal microscopy and western blotting independently (Fig 6B). The drug susceptibility revealed that only four of the seven conserved residues (G187A, P189A, F933A and Q942A) were selectively susceptible to the tested drugs, while other three equally conserved residues (G192A, D944A and H946A) either did not show any impact on drug susceptibility or were only marginally susceptible to some drugs (Fig 6C). The four drug susceptible mutants (G187A, P189A, F933A and Q942A) elicited ATPase activities similar to WT protein (Fig 6D). While most of the mutants displayed no considerable defect in R6G transport, the two drug susceptible mutants from Q-loop region showed up to 40 percent reduction in R6G transport (Fig 6D).

Considering the phenotypic behavior of these mutations flanking the two suppressors (G190R & R935T), it became apparent that these residues were not involved in the ATP catalytic cycle and thus were more likely to be involved in inter domain communication between ICL1 and NBDs. This also emphasizes the fact that these NBD regions selectively have communication roles similar to that of the drug susceptible residues from ICLs.

## Discussion

The *C. albicans* ABC transporter Cdr1p is a major membrane transporter responsible for the emergence of multidrug resistance because of its well-known ability to export a wide variety of structurally unrelated drugs and xenobiotics^3^,^9^. Cdr1p utilizes membrane-confined TMDs for drug binding and efflux while its cytosolic NBD domains use ATP hydrolysis to power the drug extrusion. The coupling of ATP hydrolysis to drug transport involving different domains represents a critical step of the transport cycle of ABC transporters. Characterizing the coupling interface in a functional protein, however, has been challenging. We examined ICL residues in the yeast ABC transporter Cdr1p to define an interface that is most likely involved in the coupling process.

To identify residues involved in inter domain signaling, the entire primary sequence of Cdr1p ICLs was subjected to alanine scanning mutagenesis, replacing individual amino acid residues with an alanine. Phenotypic and biochemical mapping of all Cdr1p ICL mutant variants provided new insights into the coupling between drug transport and nucleotide hydrolysis. For example, though most ICL residues resulted in neutral mutations with phenotypes comparable to the WT protein, several critical residues were scattered across different ICLs that caused drug susceptibility when replaced with alanine (Supplementary Fig. S1A–D). Furthermore, the most highly conserved residues were the least important, as evidenced by their neutral phenotypes when replaced with alanine. Thus, high conservation scores of individual ICL amino acids do not necessarily indicate functional relevance. The drug hyper susceptibility pattern of critical residues showed high variability suggesting that the impact of ICL mutations was not substrate specific.

Drug-susceptible ICL mutants that do not have expression and localization defects also generally possess normal ATPase activity. Of note, we could not measure the efflux due to the non- availability of fluorescent/radiolabeled xenobiotic compounds against which ICL mutants displayed enhanced susceptibility; however, considering the enhanced drug sensitivity of ICL mutants to xenobiotics, the efflux of these compounds is expected to be defective. This result would indicate that the near normal ATPase activity is uncoupled from drug efflux in the drug-susceptible ICL mutants. In this context two ICL1 mutants, I574A and S593A against which respective chromosomal suppressors were recovered, require special mention. The mutant variant S593A demonstrated a slightly reduced ATPase activity albeit with no major changes in the kinetic parameters of ATP catalysis. The other mutant I574A was similar to the S593A mutation, except that it displayed near normal ATPase activity. Considering that we were able to recover chromosomal suppressors against both these mutants in NBDs (discussed later), the reduced drug transport and drug resistance were more likely due to disruption of inter-domain signaling rather than due to fluctuations in ATPase activity.

Contrary to some ABC transporters from higher eukaryotes^21^,^22^,^25^,^26^,^27^,^31^, Cdr1p ICL residues cause drug susceptibility upon replacement with alanine, but their phenotypes are not generally linked to defects in protein folding and/or recruitment to the PM. Our data indicate that related phenotypic changes due to ICL1 mutations are caused by the disruption of the communication interface rather than a direct effect on drug transport. This communication role of ICL residues was reaffirmed by the recovery of spontaneous chromosomal suppressors against the drug-susceptible ICL mutants.

Our strategy to find second site mutations that reverse drug susceptibility led to the isolation of two suppressor mutations against two drug-susceptible ICL1 mutants. The G190R and R935T suppressor mutations which resulted in charge modification, were recovered from ICL1 mutants S593A and I574A, respectively, and were able to restore drug resistance comparable to WT cells. The localization of both these chromosomal suppressors within the NBDs emphasizes the importance of the ICL-NBD interface critical for the communication between the NBD and TMD domains via ICLs. The G190R suppressor mutation falls within the Walker A motif of NBD1 (VLGRP**G**AGCST) while R935T maps near the Q-loop in NBD2 (DSSFQ**R**SIGYV). Our results are consistent with earlier studies showing that residues important for inter domain communication in BtuCD and Sav1866 fall around the Q loop or X loop of NBD domains^16^,^32^. This evidence is supported and validated by many structural, genetic and mutational studies that have also provided evidence of the involvement of ICLs in inter domain communication in bacterial and human ABC transporters^33^,^34^,^35^,^36^,^37^.

The yeast ABCG transporter Pdr5p apparently also retains this feature and show that the ICL-NBD interface represents a coupling surface between NBD and TMD domains. Suppressor mutations recovered against TMS2 mutant are predominantly located either in or near the Q-loop of NBD1 or in the corresponding ICL1 residue, thus suggesting the importance of the ICL-NBD interface for communication. Residues from Q-loop regions are considered non-essential for ATP catalysis but their replacement upon mutation disrupt drug transport due to their role in signaling between NBDs and TMDs^18^,^19^.

**Figure 7:**
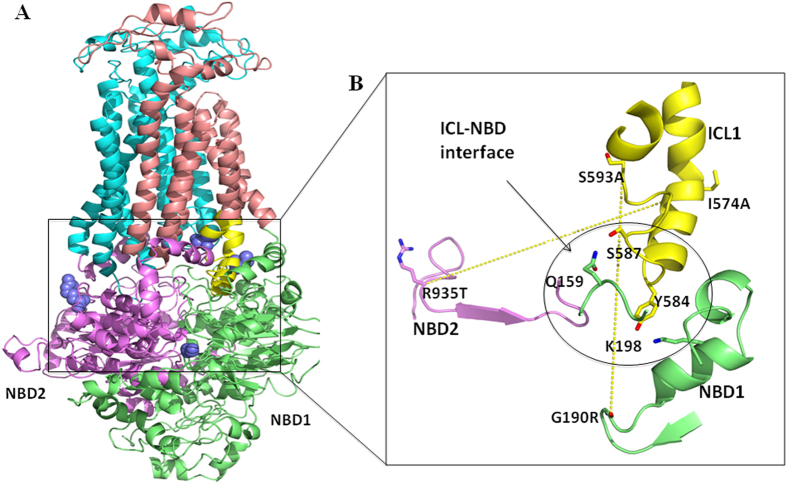
Homology model of *C. albicans* ABC drug transporter Cdr1p based on Pdr5p model 46. **A**) The TMD and NBD domains of Cdr1p are shown in distinct colors. The ICL1 loop connecting the TMS2 and TMS3 helices is shown in yellow. The ICL1 mutations I574A and S593A and corresponding suppressor mutations R935T in NBD2 and G190R in NBD1 are shown in blue. **B**) Magnification of the communication interface illustrates the location of the R935T, the Cdr1p I574A suppressor mutant, and G190R, a suppressor mutant of Cdr1p S593A. The residues involved in direct contacts between ICL1 and NBD1 are encircled. The dotted lines (in yellow) denote the distance between the two ICL residues and their respective chromosomal suppressors.

We used homology models of Cdr1p in ATP bound (closed) and ATP-unbound (open) forms^23^,^38^ to examine the possible interactions between drug-susceptible ICL1 mutants and their suppressor mutants in the Cdr1p NBDs (Fig. 7A). The I574A mutation falls close to TMS2, and its suppressor mutant R935T falls near the Q loop of NBD2. The S593R mutation falls close to the TMS3 helix and its suppressor mutant G190R is located in the NBD1 Walker A motif (Fig. 7B).

**Figure 8:**
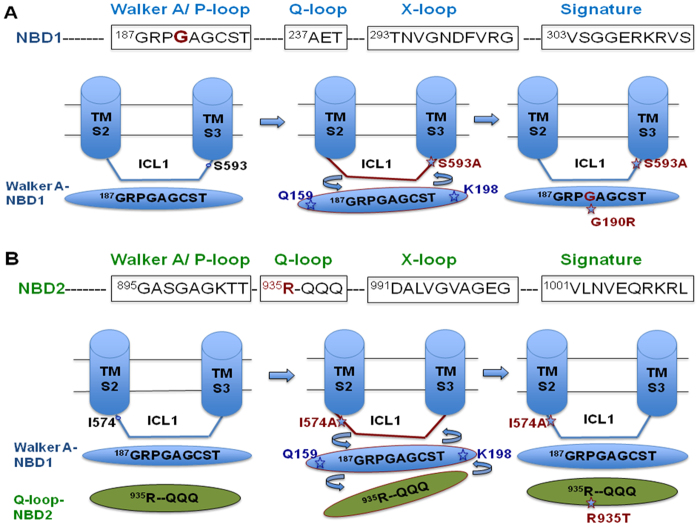
Schematic hypothetical model of coupling interface between drug-susceptible ICL1 mutants and their suppressor mutants in NBDs of Cdr1p. **A**) The drug-susceptible S593A mutation in the central panel induces the conformational change in ICL1 loop that perturbs the physiological coupling between ATPase activity and substrate transport. The suppressor mutant G190R in NBD1 Walker A motif orients NBD1 and re-establishes the physiological contacts between NBD1 and ICL1 of Cdr1p. **B**) The drug-susceptible I574A ICL1 mutation also induces the conformational change that perturbs the physiological contacts between NBD and ICL1 of Cdr1p and impairs coupling. The suppressor mutant R935T in Q-Loop of NBD2 triggers the conformational change in ATPase binding pocket enclosed between NBD1 and NBD2, which restores the proper coupling interface between ICL1 and NBD.

The 3D model of the transporter makes it clear that drug susceptible ICL residues along with their respective suppressor mutations are located at quite a distance from each other. The distance between ICL mutant I574A and its chromosomal suppressor R935T, which is located near the Q loop of NBD2, is 62 Å, while the distance between S593A and its chromosomal suppressor G190R, which lies in sub-domain Walker A of NBD1, is 28 Å in our model, thus excluding the possibility of direct interactions between these residues (Fig. 7B). This model implies that the phenotypic reversal in the chromosomal suppressors could be the result of restoration of long distance communication indirectly. In closed Cdr1p structure, the G190 mutation in the Walker-A motif lies close to the K198 residue, which interacts directly with Y584 in the ICL1 loop (Fig 7B). The S593A mutation in the ICL1 loop results in a conformational change that disrupts the coupling interface between the TMD and NBD of Cdr1p. The G190R suppressor mutation in the Walker A motif of NBD1 reestablishes the contacts between NBD1 and ICL1, which reverses the phenotypic affect of S593A mutation (Fig. 8A). For the G190R mutation within the Walker A motif, arginine-190 has more rotamers, which results in an increased conformational flexibility that restores the proper contacts between ICL1 and NBD1 in the suppressor mutant (S593A/G190R) of Cdr1p. The current results show that ICL1 and NBD1 are strongly coupled via specific buried communication interface between them. Notably, the Sav1866 crystal structure shows that the region between Walker A and signature motif of NBD1 (known as X- loop) is also found to interact directly with ICL1 loop of the protein^16^.

In the closed Cdr1p structure, ATP is sandwiched between the Walker A and B motifs, Q-loop and the H-loop of NBD1 against signature sequence and D-loop of NBD2. The asymmetry between NBD1 and NBD2 active sites is similar to that observed in most members of the ABC transporter family. The second drug-susceptible I574A Cdr1p ICL1 mutant also induces a conformational change that uncouples the NBD and transport substrate site (Fig. 8B). We recovered R935T suppressor mutation near Q loop of NBD2, which was able to reestablish proper contacts between NBD1 and ICL1 (Fig. 8B). Though Cdr1p ICL1 does not interact directly with NBD2, the R935T mutation is able to restore the coupling between ICL1 and the NBD1. R935T mutation in Q loop of NBD2, which is likely to cause significant conformational changes in NBD1 that, establishes the proper TMD-NBD coupling, reversing the phenotypic effect of the ICL1 I574A mutation. In our study, we have identified that the drug-susceptible mutations in ICL1 are compensated by mutations in the Walker A motif of NBD1 and Q loop of NBD2 of Cdr1p. Organization of TMD and NBD and key residues involved in TMD-NBD coupling interface of Cdr1p may differ significantly from the Sav1866 crystal structure. However, the region involved in coupling ATP hydrolysis to substrate translocation is identical in both the Cdr1p model and the Sav1866 crystal structure.

In conclusion, this study examines the inter domain communication role of all the ICL residues of a yeast ABC transporter Cdr1p for the first time. We identified two ICL1 mutants that interact with chromosomal suppressor mutations in NBDs through long-range interactions. Additionally, we could also recover a suppressor (W1038L) against K577A drug susceptible ICL1 mutant and four suppressors (L1032F, C1041W, R911I, R1008I) against E597A (Supplementary Table S3). Interestingly, most of these suppressor mutations also fall in the D-loop of NBD2 which lies next to Q-loop region. This further, reinforces the importance of the D-loop and Q-loop regions for maintaining the communication between NBD and TMD regions of Cdr1p. Q-loop and/or D-loop mutations have also been previously well documented in Pdr5p, Pgp and other ABC transporters to have a significant role in maintaining inter domain communication and drug resistance without significantly affecting ATP catalytic cycle^21^,^37^,^39^. It is worth mentioning that the D-Loop region along with the Signature sequence motif of NBD2 also form the ATP sandwich site along with Walker A, Walker B and Q-loop region of NBD1^40^, thus highlighting the importance of this region in multiple communications.

**Figure 9:**
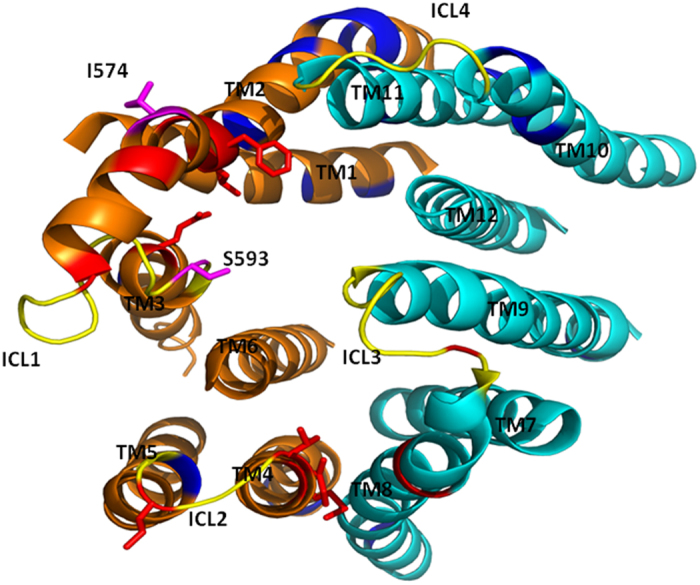
View of the Cdr1p TMDs in closed conformation from cytoplasm. All Cdr1p ICLs are shown in yellow. All total drug susceptible mutations determined previously^23^ in TMDs are shown in blue with TMS1-6 (designated as TM1-6) shown in orange while TMS7-12 (designated as TM7-12) shown in cyan. Two drug-susceptible ICL1 mutations, I574 and S593, which have suppressor mutations in NBDs, are shown in pink. Other drug-susceptible ICL residues in stick model are shown in red and may be involved directly in drug binding interactions of Cdr1p.

Our study also suggests that not all ICL residues have a direct role in interfacial communication. Because ICLs are the extensions of TMSs, and many of the ICL residues located at these cytosolic extensions also fall closer to the drug binding pocket, they could thus impact the binding or release of the drug directly rather than acting as interfacial residues (Fig. 9). In particular, this phenomenon may be possible in drug-susceptible mutants, as depicted in the deduced stick model (red in Fig. 9) in which, the faces are closer to drug binding pocket. Therefore, the loss of transport ability in these variants could be due to a defect in the binding or release of the drug rather than the disruption of inter-domain communication. This possibility needs to be examined in more detail. Finally, identifying uncoupling in a system where the binding of drugs does not cause an enhanced rate of ATPase activity, perhaps due to the presence of endogenous substrates, still remains a challenge. Nevertheless, this study provides an initial insight into the role of ICLs in the activity of a medically important ABC transporter Cdr1p, which could help to improve therapeutic strategies against *Candida* infections.

## Methods

### Materials

R6G, KTC, ANI, MIC, CTR, CYH, ITC, NR, adenosine triphosphate (ATP), oligomycin (OM), trypsin, phenylmethanesulfonyl fluoride (PMSF), p-tosyl-L-lysine chloromethyl ketone (TLCK), and tosyl phenylalanyl chloromethyl ketone (TPCK) were procured from Sigma Chemical Co. (St. Louis, MO). The protease inhibitors leupeptin, pepstatin A, and aprotinin were purchased from G-Biosciences (MO, USA). FLC was generously provided by Ranbaxy, India. Ascorbic acid (AA) was purchased from SRL (Mumbai, India). Oligonucleotides used in this study were commercially procured from Sigma Genosys, India and are listed in Supplementary Table S4. Anti-GFP monoclonal antibody and anti-mouse secondary antibody were purchased from Santa Cruz Biotechnology Inc. (Texas, USA). The anti-Pma1 (PM ATPase) polyclonal antibody was a gift from Professor Ramon Serrano (Universidad Politecnica de Valencia-CSIC, Valencia, Spain). ^3^H-radiolabeled FLC (^3^H-FLC) was purchased from Moravek Biochemicals and Radiochemicals (CA, USA).

### Strains and media components used

The yeast strains used in the study are listed in Supplementary Table S5. Plasmids were maintained in *E. coli* Dh5α strain cultured in Luria-Bertani medium (Difco, BD Biosciences, MD, USA) to which ampicillin was added (0.1 mg/ml). WT and *CDR1* mutant strains were cultured either in YEPD broth or on YEPD agar plates. Media chemicals were obtained either from Difco (Detroit, MI) or HiMedia (Mumbai, India). SD-ura- dropout medium (0.67% yeast nitrogen base, 0.2% dropout mix and 2% glucose) containing 2.5% (w/v) agar was used for growth and selection of mutants after yeast integration.

### Drug susceptibility assays

Susceptibility to various drugs was evaluated either by broth microdilution or serial dilution spot assay. Cell suspensions were fivefold serially diluted in saline solution, and a 4 μl aliquot was spotted on drug-containing or untreated YEPD plates as described previously^24^,^41^.

### Site directed mutagenesis

Introduction of mutations at particular position was achieved by PCR amplification of the pPSCDR1-GFP plasmid containing the *CDR1* gene with pre-designed primers containing the mutation of interest. Mutagenesis was performed by Quick Change site directed mutagenesis kit from Agilent technologies as per the manufacturer’s instructions. The introduction of mutations was confirmed by DNA sequencing. Positive plasmid clones were used for transformation of the *S. cerevisiae* strain AD1-8u- using the lithium acetate transformation protocol after linearization with XbaI as described previously^41^.

### Immunodetection and Confocal microscopy

PM was prepared and used for immunodetection of Cdr1 protein in WT and mutant strains as described previously^42^. Immunodetection of GFP-tagged Cdr1 protein was performed using HRP-labeled anti-GFP antibody at a 1:5000 dilution. Pma1p (PM ATPase1) was used as loading control and was probed using anti-pma1polyclonal antibody (1:5000 dilution). GFP-tagged proteins were imaged using Olympus FluoView™ FV1000 laser confocal microscope (PA, USA) with 100X oil immersion objective lens.

### Substrate transport assays

R6G efflux was determined essentially as described previously^42^. Briefly, log phase cells were washed and resuspended as a 2% suspension in PBS solution and incubated for 2 h in the presence of R6G at a10 μM final concentration. After washing, the cells were resuspended in PBS with 2% glucose. After 40 min, a 1 ml aliquot was centrifuged, and the absorbance of the supernatant was measured at 527 nm. NR was used at a final concentration of 7 μM, and its accumulation in WT and *CDR1* mutants was assessed by flow cytometry using a FACSort flow cytometer (Becton–Dickinson Immunocytometry Systems, San Jose, CA). CellQuest software (Becton Dickinson Immunocytometry Systems, San Jose, CA) was used for data analysis^43^. Accumulation of radiolabelled FLC (^3^H-FLC) was performed as described previously^24^. Briefly, mid log phase cells were centrifuged and washed with PBS at 4000 rpm for 5 min. After de-energization for 2 h, 0.5 ml of 5% cell suspension in YNB was incubated with 100 nM final concentration of ^3^H-FLC in the presence of 2% glucose. For each WT and mutant cell suspension, a 100 μl aliquot was taken after 45 min of incubation at 30 °C, filtered through a 0.45 μm cellulose nitrate filter using millipore manifold filtration assembly and washed twice with PBS. The dried filter discs immersed in liquid scintillation cocktail mixture were used to measure the accumulated radioactivity using liquid scintillation counter (Tri-Carb 2900TR Liquid Scintillation Analyzer; Packard).

### OM-sensitive ATPase assay

ATPase activity due to Cdr1p in WT and mutant variants was monitored by the oligomycin-sensitive release of inorganic phosphate as described previously^42^. 10 μg protein from WT and each mutant strain PM fraction was used to determine the Cdr1p ATPase activity in an *in-vitro* assay. The reaction was started by adding 5 mM ATP to the reaction mixture and was terminated after 30 min of incubation at 30 °C by addition of 1 ml of stop solution containing 0.5% SDS.

For the calculations of protein specific activities for kinetic parameters, the amounts of Cdr1-GFP in membrane protein fraction in WT and mutant strains were calculated using Dot-blot assay. Known amounts of GFP were used to obtain a linear calibration data set to calculate the amount of Cdr1-GFP in membrane fractions from individual strains. The Cdr1-protein amount obtained above was used to calculate the concentration of protein in nmoles which in turn was used to calculate the kcat values using the Vmax values obtained for the same protein. Within the sensitivity limits of the method, we believe we have a fair estimate of the amount of Cdr1-GFP in the crude membrane preparations.

### Protease protection assay

Limited proteolysis was performed by digesting PM fractions that contained CDR1-GFP or mutant protein variants (40 μg) with a protein:enzyme ratio of 20:1 with purified trypsin in 50 mM Tris-HCl, pH 7.5^28^,^44^. The digests were incubated at 4 °C for 5 min, and the reactions were stopped by adding PMSF at final concentration of 1 mM and TLCK at a final concentration of 5 mM followed by incubation for 10 min on ice. The samples were denatured by the addition of 5X SDS sample buffer, resolved on 8% polyacrylamide gels followed by transfer on PVDF/nitrocellulose membranes^45^. Western blotting was performed against GFP-tagged protein as described above.

### Generation of suppressor strains and their sequence analysis

Cells from overnight cultures were mixed with the warm agar to final OD of 10^5^ cells, poured and grown in the presence of toxic concentrations of different drugs, such as FLC (20 μg), KTC (1 μg), and CYH (1 μg), on filter discs for prolonged periods at 30 °C as described previously^38^. Agar plates were monitored for the appearance of colonies at 6-7 days within the drug inhibitory zones. These colonies were picked and further analyzed for acquisition of spontaneous mutations. The strains with spontaneous mutations that retained drug resistant phenotype after several passages were selected, and their genomic DNA was isolated. The whole *CDR1* gene was sequenced from amplicons containing the *CDR1* orf amplified from each suppressor strain genomic DNA. Sequencing was repeated at least three times with overlapping primers to minimize the errors incurred during sequencing. The final base changes observed was translated to amino acid substitutions acquired by the Cdr1p protein.

## Additional Information

**How to cite this article**: Shah, A. H. *et al.* Mutational Analysis of Intracellular Loops Identify Cross Talk with Nucleotide Binding Domains of Yeast ABC Transporter Cdr1p. *Sci. Rep.* **5**, 11211; doi: 10.1038/srep11211 (2015).

## Supplementary Material

Supplementary Information

## Figures and Tables

**Table 1:** MIC_80_ values of different drug-susceptible mutants obtained by broth micro dilution assay.

	**ANI**	**CTR**	**CYH**	**FLC**	**ITR**	**KTC**	**MIC**	**R6G**
**AD 1-8u**^**−**^	0.125	.02	0.008	0.5	0.062	0.004	0.008	.2
**CDR1-GFP**	8	2	1	64	32	0.5	1	32
**L568A**	4	2	0. 25	8	4	0.062	0.015	4
**F569A**	4	2	0.25	8	32	0.062	0.015	8
**E570A**	2	0.5	0.125	8	1	0.031	0.015	8
**I574A**	2	0.25	0.062	2	0.5	0.031	0.008	4
**K577A**	2	0.125	0.062	8	0.5	0.031	0.031	8
**L583A**	8	0.25	0.125	32	32	0.062	0.062	16
**S593A**	1	0.125	0.015	1	0.25	0.004	0.015	4
**E597A**	1	0.125	0.015	1	0.25	0.004	0.015	1
**I646A**	1	0.125	0. 25	8	2	0.125	0.062	4
**V649A**	2	0.5	0. 25	8	2	0.031	0.125	8
**I653A**	2	0.25	0. 25	8	1	0.031	0.015	2
**M657A**	1	0.125	0.125	4	1	0.031	0.008	8
**Y1249A**	2	0.5	0.25	16	4	0.125	0.25	8
**F1250A**	4	1	0.125	16	16	0.125	0.031	8
**V1259A**	4	0.5	0.125	16	4	0.125	0.031	4

MICs of all ICL mutants were determined by broth micro dilution method as described in methods and in our earlier publications^24^. The table depicts MICs of mutants showing susceptibility to two or more drugs.

**Table 2:** Kinetics of ATPase activities and drug transport of the drug-susceptible mutants I574A, S593A and their respective chromosomal suppressor strains

**A**				
	**V_max_ (nmole min^−1^ mg^−1^)**	**K_m_ (mM)**	**k_cat_ (s^−1^)**	**k_cat_/K_m_(s^−1^mM^−1^)**
**CDR1-GFP**	113.7 ± 10.9	5.69 ± 0.79	48.2*10^3^ ± 1.1	7.78*10^3^ ± 0.23
**I574A**	85.6 ± 7.2	6.06 ± 1.36	56.2*10^3^ ± 3.4	8.41*10^3^ ± 0.82
**I574A/R**	94.4 ± 9.8	5.73 ± 1.71	62.9*10^3^ ± 1.1	10.01*10^3^ ± 2.31
**S593A**	65.3 ± 1.7	3.39 ± 0.23	47.1*10^3^ ± 2.8	13.19*10^3^ ± 0.42
**S593A/R**	84.5 ± 5.5	6.34 ± 0.55	55.7*10^3^ ± 1.5	8.38*10^3^ ± 0.55

**B**				
	**A_max_ (pmole/mg dry wt.) [^3^H-FLC accumulation]**		**K_d_ (nM) [^3^H-FLC Binding]**	
**CDR1-GFP**	0.160 ± 0.01		0.148 ± 0.03	
**I574A**	0.322 ± 0.02		0.329 ± 0.04	
**I574A/R**	0.176 ± 0.02		0.124 ± 0.01	
**S593A**	0.279 ± 0.03		0.278 ± 0.08	
**S593A/R**	0.134 ± 0.01		0.131 ± 0.01	

A) ATPase activities of the mutants compared to WT Cdr1p; the mutants showing considerable change in activity are underlined. k_cat_ values are calculated per mg of Cdr1 protein. B) ^3^H-FLC transport kinetics measured as the radiolabelled ^3^H-FLC accumulated inside the cells. The mutants showing a considerable change in drug accumulation inside the cells are underlined. The values are the average ± S.D. of at least three independent experiments.

## References

[b1] Richardson M. D. Changing patterns and trends in systemic fungal infections. *J Antimicrob Chemother*. 56, i5–i11 (2005). 16120635 10.1093/jac/dki218

[b2] Prasad R., Shah A. H. & Dhamgaye S. Mechanisms of drug resistance in fungi and their significance in biofilms. *Antibiofilm Agents, Springer Series on Biofilms*. 8, 45–65 (2014).

[b3] White T C., Holleman S., Dy F., Mirels L. F. & Stevens D. A. Resistance mechanisms in clinical isolates of *Candida albicans*. *Antimicrob Agents Chemother*. 46, 1704–1713 (2002). 12019079 10.1128/AAC.46.6.1704-1713.2002 PMC127245

[b4] White T. C., Marr K. A. & Bowden R. A. Clinical, cellular, and molecular factors that contribute to antifungal drug resistance. *Clin Microbiol Rev*. 11, 382–402 (1998). 9564569 10.1128/cmr.11.2.382 PMC106838

[b5] Prasad R., De Wergifosse P., Goffeau A. & Balzi E. Molecular cloning and characterization of a novel gene of *Candida albicans*, *CDR1* conferring multiple resistance to drugs and antifungals. *Curr Genet*. 27, 320–329 (1995). 7614555 10.1007/BF00352101

[b6] Sanglard D., Ischer F., Monod M. & Bille J. Cloning of *Candida albicans* genes conferring resistance to azole antifungal agents: characterization of *CDR2*, a new multidrug ABC transporter gene. *Microbiology*. 143, 405–416 (1997). 9043118 10.1099/00221287-143-2-405

[b7] Goldway M., Teff D., Schmidt R., Oppenheim A. B. & Koltin Y. Multidrug resistance in *Candida albicans*: disruption of the BENr gene. *Antimicrob Agents Chemother*. 39, 422–426 (1995). 7726508 10.1128/aac.39.2.422 PMC162553

[b8] Pasrija R., Banerjee D. & Prasad R. Structure and function analysis of CaMdr1p, a major facilitator superfamily antifungal efflux transporter protein of *Candida albicans*: identification of amino acid residues critical for drug/H+ transport. *Eukaryot Cell*. 6, 443–453 (2007). 17209122 10.1128/EC.00315-06 PMC1828935

[b9] Holmes A. R. *et al.* ABC transporter Cdr1p contributes more than Cdr2p does to fluconazole efflux in fluconazole-resistant *Candida albicans* clinical isolates. *Antimicrob Agents Chemother*. 52, 3851–3862 (2008). 18710914 10.1128/AAC.00463-08 PMC2573144

[b10] Prasad R. & Goffeau A. Yeast ATP-binding cassette transporters conferring multidrug resistance. *Annu Rev Microbiol*. 66, 39–63 (2012). 22703054 10.1146/annurev-micro-092611-150111

[b11] Cannon R. D. *et al.* Efflux-mediated antifungal drug resistance. *Clin Microbiol Rev*. 22, 291–321 (2009). 19366916 10.1128/CMR.00051-08 PMC2668233

[b12] Ernst R., Kueppers P., Stindt J., Kuchler K. & Schmitt L. Multidrug efflux pumps: substrate selection in ATP-binding cassette multidrug efflux pumps--first come, first served? *FEBS J*. 277, 540–549 (2010). 19961541 10.1111/j.1742-4658.2009.07485.x

[b13] Prasad R., Sharma M. & Rawal M. K. Functionally Relevant Residues of Cdr1p: A Multidrug ABC Transporter of Human Pathogenic *Candida albicans*. *J Amino Acids*. 2011, 531412 (2011). 22312462 10.4061/2011/531412 PMC3268037

[b14] Lamping E. *et al.* Fungal PDR transporters: Phylogeny, topology, motifs and function. Fungal Genet Biol. 47, 127–142 (2010). 19857594 10.1016/j.fgb.2009.10.007 PMC2814995

[b15] Aller S. G. *et al.* Structure of P-glycoprotein reveals a molecular basis for poly-specific drug binding. Science . 323, 1718–1722 (2009). 19325113 10.1126/science.1168750 PMC2720052

[b16] Dawson R. J. & Locher K. P. Structure of a bacterial multidrug ABC transporter. Nature . 443, 180–185 (2006). 16943773 10.1038/nature05155

[b17] Jin M.S., Oldham M.L., Zhang Q. & Chen J. Crystal structure of the multidrug transporter P-glycoprotein from *Caenorhabditis elegans*. Nature . 490, 566–569 (2012). 23000902 10.1038/nature11448 PMC3482266

[b18] Sauna Z. E. *et al.* Mutations define cross-talk between the N-terminal nucleotide-binding domain and transmembrane helix-2 of the yeast multidrug transporter Pdr5: possible conservation of a signaling interface for coupling ATP hydrolysis to drug transport. *J Biol Chem*. 283, 35010–35022 (2008). 18842589 10.1074/jbc.M806446200 PMC2596398

[b19] Ananthaswamy N. *et al.* The signalling interface of the yeast multidrug transporter Pdr5 adopts a cis conformation, and there are functional overlap and equivalence of the deviant and canonical Q-loop residues. *Biochemistry*. 49, 4440–4449 (2012). 20426485 10.1021/bi100394j PMC2954458

[b20] Downes M. T. *et al.* The transmission interface of the Saccharomyces cerevisiae multidrug transporter Pdr5: Val-656 located in intracellular loop 2 plays a major role in drug resistance. *Antimicrob Agents Chemother*. 57, 1025–1034 (2013). 23254431 10.1128/AAC.02133-12 PMC3553689

[b21] Pagant S., Brovman E. Y., Halliday J. J. & Miller E. A. Mapping of interdomain interfaces required for the functional architecture of Yor1p, a eukaryotic ATP-binding cassette (ABC) transporter. *J Biol Chem*. 283, 26444–26451(2008). 18644782 10.1074/jbc.M803912200 PMC2546530

[b22] Pagant S., Halliday J. J., Kougentakis C. & Miller E. A. Intragenic suppressing mutations correct the folding and intracellular traffic of misfolded mutants of Yor1p, a eukaryotic drug transporter. *J Biol Chem*. 285, 36304–36314 (2010). 20837481 10.1074/jbc.M110.142760 PMC2978558

[b23] Rawal M. K. *et al.* Insight into pleiotropic drug resistance ATP-binding cassette pump drug transport through mutagenesis of Cdr1p transmembrane domains. *J Biol Chem*. 288, 24480–24493 (2013). 23824183 10.1074/jbc.M113.488353 PMC3750147

[b24] Mukhopadhyay K., Kohli A. & Prasad R. Drug susceptibilities of yeast cells are affected by membrane lipid composition. *Antimicrob Agents Chemother*. 46, 3695–3705 (2002). 12435664 10.1128/AAC.46.12.3695-3705.2002 PMC132749

[b25] Seibert F. S. *et al.* Disease-associated mutations in cytoplasmic loops 1 and 2 of cystic fibrosis transmembrane conductance regulator impede processing or opening of the channel. Biochemistry 36, 11966–11974 (1997). 9305991 10.1021/bi9712652

[b26] Iram S. H. & Cole S. P. Expression and function of human *MRP1* (ABCC1) is dependent on amino acids in cytoplasmic loop 5 and its interface with nucleotide binding domain 2. *J Biol Chem*. 286, 7202–7213 (2011). 21177244 10.1074/jbc.M110.166959 PMC3044977

[b27] Kapoor K., Bhatnagar J., Chufan E. E. & Ambudkar S. V. Mutations in intracellular loops 1 and 3 lead to misfolding of human P-glycoprotein (ABCB1) that can be rescued by cyclosporine A, which reduces its association with chaperone Hsp70. *J Biol Chem*. 288, 32622–32636 (2013). 24064216 10.1074/jbc.M113.498980 PMC3820894

[b28] Mandal A. *et al.* A key structural domain of the *Candida albicans* Mdr1 protein. *Biochem J*. 445, 313–322 (2012). 22587419 10.1042/BJ20120190

[b29] Jha S. *et al.* Purification and characterization of the N-terminal nucleotide binding domain of an ABC drug transporter of *Candida albicans*: uncommon cysteine 193 of Walker A is critical for ATP hydrolysis. Biochemistry 42, 10822–10832 (2003). 12962507 10.1021/bi0345900

[b30] Jha S., Karnani N., Lynn A. M. & Prasad R. Covalent modification of cysteine 193 impairs ATPase function of nucleotide-binding domain of a Candida drug efflux pump. *Biochem Biophys Res Commun*. 310, 869–875 (2003). 14550284 10.1016/j.bbrc.2003.09.094

[b31] Loo T. W., Bartlett M. C. & Clarke D. M. Human P-glycoprotein contains a greasy ball-and-socket joint at the second transmission interface. *J Biol Chem*. 288, 20326–20333 (2013). 23733192 10.1074/jbc.M113.484550 PMC3711299

[b32] Locher K. P., Lee A. T. & Rees D. C. The *E. coli* BtuCD structure: a framework for ABC transporter architecture and mechanism. Science 296,1091–1098 (2002). 12004122 10.1126/science.1071142

[b33] Oancea G. *et al.* Structural arrangement of the transmission interface in the antigen ABC transport complex TAP. *Proc Natl Acad Sci U S A*. 106, 5551–5556 (2009). 19297616 10.1073/pnas.0811260106 PMC2657591

[b34] Cotten J. F, Ostedgaard L. S, Carson M. R. & Welsh M. J. Effect of cystic fibrosis-associated mutations in the fourth intracellular loop of cystic fibrosis transmembrane conductance regulator. *J Biol Chem*. 271, 21279–21284 (1996). 8702904 10.1074/jbc.271.35.21279

[b35] Currier S. J. *et al.* Identification of residues in the first cytoplasmic loop of P-glycoprotein involved in the function of chimeric human MDR1-MDR2 transporters. *J Biol Chem*. 267, 25153–25159 (1992). 1360983

[b36] Jones P. M. & George A. M. The ABC transporter structure and mechanism: perspectives on recent research. *Cell Mol Life Sci*. 61, 682–699 (2004). 15052411 10.1007/s00018-003-3336-9 PMC11138499

[b37] Urbatsch I. L., Gimi K., Wilke-Mounts S. & Senior A. E. Investigation of the role of glutamine-471 and glutamine-1114 in the two catalytic sites of P-glycoprotein. Biochemistry. 39, 11921–11927 (2000). 11009605 10.1021/bi001220s

[b38] Niimi K. *et al.* Specific interactions between the *Candida albicans* ABC transporter Cdr1p ectodomain and a D-octapeptide derivative inhibitor. *Mol Microbiol*. 85, 747–767 (2012). 22788839 10.1111/j.1365-2958.2012.08140.x PMC3418399

[b39] Furman C. *et al.* The deviant ATP-binding site of the multidrug efflux pump Pdr5 plays an active role in the transport cycle. *J Biol Chem*. 288, 30420–30431 (2013). 24019526 10.1074/jbc.M113.494682 PMC3798506

[b40] Sauna Z. E. & Ambudkar S. V. About a switch: how P-glycoprotein (ABCB1) harnesses the energy of ATP binding and hydrolysis to do mechanical work. Mol. Cancer Ther. 6, 13–23 (2007). 17237262 10.1158/1535-7163.MCT-06-0155

[b41] Shah A. H. *et al.* Novel role of a family of major facilitator transporters in biofilm development and virulence of *Candida albicans*. *Biochem. J*. 460, 223–235 (2014). 24621232 10.1042/BJ20140010

[b42] Shukla S. *et al.* Functional characterization of *Candida albicans* ABC transporter Cdr1p. *Eukaryot Cell*. 2, 1361–1375 (2003). 14665469 10.1128/EC.2.6.1361-1375.2003 PMC326652

[b43] Ivnitski-Steele I. *et al.* Identification of Nile red as a fluorescent substrate of the *Candida albicans* ATP-binding cassette transporters Cdr1p and Cdr2p and the major facilitator superfamily transporter Mdr1p. *Anal Biochem*. 394, 87–91(2009). 19577533 10.1016/j.ab.2009.07.001 PMC2739806

[b44] Ndayizeye M., Touret N. & Fliege L. Proline 146 is critical to the structure, function and targeting of sod2, the Na+/H+ exchanger of *Schizosaccharomyces pombe*. *Biochim Biophys Acta*. 1788, 983–992 (2009). 19171118 10.1016/j.bbamem.2009.01.001

[b45] Landolt-Marticorena C., Williams K. M., Correa J., Chen W. & Manolson M. F. Evidence that the NH2 terminus of vph1p, an integral subunit of the V0 sector of the yeast V-ATPase, interacts directly with the Vma1p and Vma13p subunits of the V1 sector. *J Biol Chem*. 275, 15449–15457 (2000). 10747882 10.1074/jbc.M000207200

[b46] Rutledge R. M., Esser L., Ma J. & Xia D. Toward understanding the mechanism of action of the yeast multidrug resistance transporter Pdr5p: a molecular modeling study. *J Struct Biol*. 173, 333–344 (2011). 21034832 10.1016/j.jsb.2010.10.012 PMC3026082

