# Supplementary material for: Mutational Analysis of Intracellular Loops Identify Cross Talk with Nucleotide Binding Domains of Yeast ABC Transporter Cdr1p
Source: Sci Rep. 2015 Jun 8;5:11211. doi: 10.1038/srep11211 (PMC4459223; doi:10.1038/srep11211)
Supplement: Supplementary Information [file srep11211-s1.pdf]

Mutational Analysis of Intracellular Loops Identify Cross Talk with Nucleotide Binding Domains of Yeast ABC Transporter Cdr1p

Abdul Haseeb Shah, Manpreet Kaur Rawal, Sanjiveeni Dhamgaye, Sneha Sudha Komath, Ajay Kumar Saxena and Rajendra Prasad

School of Life Sciences, Jawaharlal Nehru University, New Delhi 110067, India

SUPPLEMENTARY DATA

Supplementary Table S1: Cdr1p Intrcellular loop conservation scores. Conservation scores were obtained using JALVIEW2.4.0.b2 (http://www.jalview.org)

Loops	Sum of conservation scores of all columns (a)	No of Columns (n)	Loop conservation score (average score) LCS = a/n
**ICL1**	188	31	6.06
**ICL2**	73	13	5.61
**ICL3**	176	32	5.5
**ICL4**	33	9	3.6

Supplementary Table S2: MIC_{80} values of two drug susceptible ICL1 mutants and there suppressors compared to WT strain.

	CTR	CYH	FLU	ITR	KTC	MIC	R6G
**AD 1-8u-**	0.02	0.008	0.5	0.0625	0.004	0.008	0.25
**CDR1-GFP**	2	1	64	32	0.5	1	32
**I574A**	0.25	0.125	4	0.5	0.015	0.008	4
**I574A/R**	0.5	0.25	32	8	0.125	0.125	16
**S593A**	0.125	0.015	1	0. 25	0.008	0.008	4
**S593A/R**	0.5	0.5	64	8	0.5	0.25	32

Supplementary Table S3: **Summary of suppressors recovered in this study**

ICL Drug sensitive mutant	Suppressor mutation position	Drug	Location of suppressor mutation
I574A	Reversion to WT	FLC	Native position
	R935T	KTC	Near Q Loop of NBD2
K577A	W1038L	FLC	After D Loop of NBD2
S593A	G190R	KTC	Walker A of NBD1
E597A	L1032F	FLC	In D Loop of NBD2

	**C1041W**	**KTC**	**After D Loop of NBD2**
	**R911I**	**CYH**	**After Walker A of NBD2**
	**R1008I**	**CYH**	**In Signature Sequence of NBD2**

Supplementary Table S4: List of oligonucleotides used in the study:

Name	Sequence
**Primers for alanine scanning mutagenesis of ICL1 of CDR1 (31 residues) (5' to 3')**	
**CDR1 S567A/F**	CTTTTGGAAATCATGGCACTTTTCGAAGC
**CDR1 S567A/R**	GCTTCGAAAAGTGCCATGATTTCCAAAAG
**CDR1 L568A/F**	GGAAATCATGTCAGCTTTCGAAGCCAGAC
**CDR1 L568A/R**	GTCTGGCTTCGAAAGCTGACATGATTTCC
**CDR1 F569A/F**	GAAATCATGTCACTTGCCGAAGCCAGACC
**CDR1 F569A/R**	GGTCTGGCTTCGGCAAGTGACATGATTTC
**CDR1 E570A/F**	CATGTCACTTTTCGCAGCCAGACCAATTG
**CDR1 E570A/R**	CAATTGGTCTGGCTGCGAAAAGTGACATG
**CDR1 A571G/F**	GTCACTTTTCGAAGGCAGACCAATTGTC
**CDR1 A571G/R**	GACAATTGGTCTGCCTTCGAAAAGTGAC
**CDR1 R572A/F**	CACTTTTCGAAGCCGCACCAATTGTCGAG
**CDR1 R572A/R**	CTCGACAATTGGTGCGGCTTCGAAAAGTG
**CDR1 P573A/F**	TTTCGAAGCCAGAGCAATTGTCGAGAAAC
**CDR1 P573A/R**	GTTTCTCGACAATTGCTCTGGCTTCGAAA
**CDR1 I574A/F**	CGAAGCCAGACCAGCTGTCGAGAAAC
**CDR1 I574A/R**	GTTTCTCGACAGCTGGTCTGGCTTCG
**CDR1 V575A/F**	GAAGCCAGACCAATTGCCGAGAAACATAAAA
**CDR1 V575A/R**	TTTTATGTTTCTCGGCAATTGGTCTGGCTTC
**CDR1 E576A/F**	CCAGACCAATTGTCGCGAAACATAAAAAATATG
**CDR1 E576A/R**	CATATTTTTTATGTTTCGCGACAATTGGTCTGG
**CDR1 K577A/F**	GACCAATTGTCGAGGCACATAAAAAATATG
**CDR1 K577A/R**	CATATTTTTTATGTGCCTCGACAATTGGTC
**CDR1 H578A/F**	CAATTGTCGAGAAAGCTAAAAAATATGCCC
**CDR1 H578A/R**	GGGCATATTTTTTAGCTTTCTCGACAATTG
**CDR1 K579A/F**	GTCGAGAAACATGCAAAATATGCCCTTTATC
**CDR1 K579A/R**	GATAAAGGGCATATTTTGCATGTTTCTCGAC
**CDR1 K580A/F**	GTCGAGAAACATAAAGCATATGCCCTTTATC
**CDR1 K580A/R**	GATAAAGGGCATATGCTTTATGTTTCTCGAC
**CDR1 Y581A/F**	GAGAAACATAAAAAAGCTGCCCTTTATCGTCC
**CDR1 Y581A/R**	GGACGATAAAGGGCAGCTTTTTTATGTTTCTC
**CDR1 A582G/F**	GAAACATAAAAAATATGGCCTTTATCGTCCTTC
**CDR1 A582G/R**	GAAGGACGATAAAGGCCATATTTTTTATGTTTC
**CDR1 L583A/F**	CATAAAAAATATGCCGCTTATCGTCCTTCAGC
**CDR1 L583A/R**	GCTGAAGGACGATAAGCGGCATATTTTTTATG
**CDR1 Y584A/F**	AAATATGCCCTTGCTCGTCCTTCAGCTG
**CDR1 Y584A/R**	CAGCTGAAGGACGAGCAAGGGCATATTT
**CDR1 R585A/F**	TATGCCCTTTATGCTCCTTCAGCTGATG

**CDR1 R585A/R**	CATCAGCTGAAGGAGCATAAAGGGCATA
**CDR1 P586A/F**	GCCCTTTATCGTGCTTCAGCTGATGCCTTG
**CDR1 P586A/R**	CAAGGCATCAGCTGAAGCACGATAAAGGGC
**CDR1 S587A/F**	CCTTTATCGTCCTGCAGCTGATGCCTTGGC
**CDR1 S587A/R**	GCCAAGGCATCAGCTGCAGGACGATAAAGG
**CDR1 A588G/F**	CTTTATCGTCCTTCAGGTGATGCCTTGGCCAG
**CDR1 A588G/R**	CTGGCCAAGGCATCACCTGAAGGACGATAAAG
**CDR1 D589A/F**	CGTCCTTCAGCTGCTGCCTTGGCCAG
**CDR1 D589A/R**	CTGGCCAAGGCAGCAGCTGAAGGACG
**CDR1 A590G/F**	GTCCTTCAGCTGATGGCTTGGCCAGTATTATTAG
**CDR1 A590G/R**	CTAATAATACTGGCCAAGCCATCAGCTGAAGGAC
**CDR1 L591A/F**	CTTCAGCTGATGCCGCGGCCAGTATTATTAG
**CDR1 L591A/R**	CTAATAATACTGGCCGCGGCATCAGCTGAAG
**CDR1 A592G/F**	CAGCTGATGCCTTGGGCAGTATTATTAGTG
**CDR1 A592G/R**	CACTAATAATACTGCCCAAGGCATCAGCTG
**CDR1 S593A/F**	CTGATGCCTTGGCCGCTATTATTAGTGAATTAC
**CDR1 S593A/R**	GTAATTCACTAATAATAGCGGCCAAGGCATCAG
**CDR1 I594A/F**	GATGCCTTGGCCAGTGCTATTAGTGAATTAC
**CDR1 I594A/R**	GTAATTCACTAATAGCACTGGCCAAGGCATC
**CDR1 I595A/F**	CTTGGCCAGTATTGCTAGTGAATTACCTG
**CDR1 I595A/R**	CAGGTAATTCACTAGCAATACTGGCCAAG
**CDR1 S596A/F**	GCCAGTATTATTGCTGAATTACCTGTC
**CDR1 S596A/R**	GACAGGTAATTCAGCAATAATACTGGC
**CDR1 E597A/F**	CAGTATTATTAGTGCATTACCTGTCAAATTAG
**CDR1 E597A/R**	CTAATTTGACAGGTAATGCACTAATAATACTG
**Primers for alanine scanning mutagenesis of ICL2 of CDR1 (13 residues) (5' to 3')**	
**CDR1 IL2 S645A/F**	CATTTGTTTAGAGCCATTGGTGCTGTTTC
**CDR1 IL2 S645A/R**	GAAACAGCACCAATGGCTCTAAACAAATG
**CDR1 IL2 I646A/F**	GTTTAGATCCGCTGGTGCTGTTTCAAC
**CDR1 IL2 I646A/R**	GTTGAAACAGCACCAGCGGATCTAAAC
**CDR1 IL2 G647A/F**	GTTTAGATCCATTGCTGCTGTTTCAACATC
**CDR1 IL2 G647A/R**	GATGTTGAAACAGCAGCAATGGATCTAAAC
**CDR1 IL2 A648G/F**	GATCCATTGGTGGTGTTTCAACATC
**CDR1 IL2 A648G/R**	GATGTTGAAACACCACCAATGGATC
**CDR1 IL2 V649A/F**	CATTGGTGCTGCTTCAACATCTATTTC
**CDR1 IL2 V649A/R**	GAAATAGATGTTGAAGCAGCACCAATG
**CDR1 IL2 S650A/F**	CATTGGTGCTGTTGCAACATCTATTTCTG
**CDR1 IL2 S650A/R**	CAGAAATAGATGTTGCAACAGCACCAATG
**CDR1 IL2 T651A/F**	GGTGCTGTTTCAGCATCTATTTCTGGTG
**CDR1 IL2 T651A/R**	CACCAGAAATAGATGCTGAAACAGCACC
**CDR1 IL2 S652A/F**	CTGTTTCAACAGCTATTTCTGGTG
**CDR1 IL2 S652A/R**	CACCAGAAATAGCTGTTGAAACAG
**CDR1 IL2 I653A/F**	GTTTCAACATCTGCTTCTGGTGCCATG
**CDR1 IL2 I653A/R**	CATGGCACCAGAAGCAGATGTTGAAAC
**CDR1 IL2 S654A/F**	CAACATCTATTGCTGGTGCCATGACTC
**CDR1 IL2 S654A/R**	GAGTCATGGCACCAGCAATAGATGTTG
**CDR1 IL2 G655A/F**	CAACATCTATTTCTGCTGCCATGACTCCTG
**CDR1 IL2 G655A/R**	CAGGAGTCATGGCAGCAGAAATAGATGTTG
**CDR1 IL2 A656G/F**	CATCTATTTCTGGTGGCATGACTCCTGC
**CDR1 IL2 A656G/R**	GCAGGAGTCATGCCACCAGAAATAGATG
**CDR1 IL2 M657A/F**	CTATTTCTGGTGCCGCGACTCCTGCTACC

**CDR1 IL2 M657A/R**	GGTAGCAGGAGTCGCGGCACCAGAAATAG
**Primers for alanine scanning mutagenesis of ICL3 of CDR1 (32 residues) (5' to 3')**	
**CDR1 IL3 P1248A/F**	CAAATGTTAGCATACTTTGTGAAG
**CDR1 IL3 P1248A/R**	CTTCACAAAGTATGCTAACATTTG
**CDR1 IL3 Y1249A/F**	AAATGTTACCAGCCTTTGTGAAGCAAC
**CDR1 IL3 Y1249A/R**	GTTGCTTCACAAAGGCTGGTAACATTT
**CDR1 IL3 F1250A/F**	GTTACCATACGCTGTGAAGCAACGTG
**CDR1 IL3 F1250A/R**	CACGTTGCTTCACAGCGTATGGTAAC
**CDR1 IL3 V1251A/F**	GTTACCATACTTTGCGAAGCAACGTGATG
**CDR1 IL3 V1251A/R**	CATCACGTTGCTTCGCAAAGTATGGTAAC
**CDR1 IL3 K1252A/F**	CATACTTTGTGGCGCAACGTGATG
**CDR1 IL3K1252A/R**	CATCACGTTGCGCCACAAAGTATG
**CDR1 IL3 Q1253A/F**	CATACTTTGTGAAGGCACGTGATGTTTATG
**CDR1 IL3 Q1253A/F**	CATAAACATCACGTGCCTTCACAAAGTATG
**CDR1 IL3 R1254A/F**	CTTTGTGAAGCAAGCTGATGTTTATGAAG
**CDR1 IL3 R1254A/R**	CTTCATAAACATCAGCTTGCTTCACAAAG
**CDR1 IL3 D1255A/F**	GTGAAGCAACGTGCTGTTTATGAAGTG
**CDR1 IL3 D1255A/R**	CACTTCATAAACAGCACGTTGCTTCAC
**CDR1 IL3 V1256A/F**	GCAACGTGATGCTTATGAAGTGAGAG
**CDR1 IL3 V1256A/R**	CTCTCACTTCATAAGCATCACGTTGC
**CDR1 IL3 Y1257A/F**	AACGTGATGTTGCTGAAGTGAGAGAAG
**CDR1 IL3 Y1257A/R**	CTTCTCTCACTTCAGCAACATCACGTT
**CDR1 IL3 E1258A/F**	CGTGATGTTTATGCAGTGAGAGAAGCTC
**CDR1 IL3 E1258A/R**	GAGCTTCTCTCACTGCATAAACATCACG
**CDR1 IL3 V1259A/F**	GTGATGTTTATGAAGCGAGAGAAGCTCC
**CDR1 IL3 V1259A/R**	GGAGCTTCTCTCGCTTCATAAACATCAC
**CDR1 IL3 R1260A/F**	GATGTTTATGAAGTGGCAGAAGCTCCATCAAG
**CDR1 IL3 R1260A/R**	GATGTTTATGAAGTGGCAGAAGCTCCATCAAG
**CDR1 IL3 E1261A/F**	GTTTATGAAGTGAGAGCAGCTCCATCAAGAAC
**CDR1 IL3 E1261A/R**	GTTCTTGATGGAGCTGCTCTCACTTCATAAAC
**CDR1 IL3 A1262G/F**	GAAGTGAGAGAAGGTCCATCAAGAACATTC
**CDR1 IL3 A1262G/R**	GAATGTTCTTGATGGACCTTCTCTCACTTC
**CDR1 IL3 P1263A/F**	GTGAGAGAAGCTGCATCAAGAACATTC
**CDR1 IL3 P1263A/R**	GAATGTTCTTGATGCAGCTTCTCTCAC
**CDR1 IL3 S1264A/F**	GAGAGAAGCTCCAGCAAGAACATTCAG
**CDR1 IL3 S1264A/R**	CTGAATGTTCTTGCTGGAGCTTCTCTC
**CDR1 IL3 R1265A/F**	GAAGCTCCATCAGCAACATTCAGTTGG
**CDR1 IL3 R1265A/R**	CCAACTGAATGTTGCTGATGGAGCTTC
**CDR1 IL3 T1266A/F**	CTCCATCAAGAGCATTCAGTTGGTTTG
**CDR1 IL3 T1266A/R**	CAAACCAACTGAATGCTCTTGATGGAG
**CDR1 IL3 F1267A/F**	CTCCATCAAGAACAGCCAGTTGGTTTGC
**CDR1 IL3 F1267A/R**	GCAAACCAACTGGCTGTTCTTGATGGAG
**CDR1 IL3 S1268A/F**	AAGAACATTCGCTTGGTTTGCATTTATTG
**CDR1 IL3 S1268A/R**	CAATAAATGCAAACCAAGCGAATGTTCTT
**CDR1 IL3 W1269A/F**	GAACATTCAGTGCGTTTGCATTTATTG
**CDR1 IL3 W1269A/R**	CAATAAATGCAAACGCACTGAATGTTC
**CDR1 IL3 F1270A/F**	CATTCAGTTGGGCTGCATTTATTGC
**CDR1 IL3 F1270A/R**	CATTCAGTTGGGCTGCATTTATTGC
**CDR1 IL3 A1271G/F**	CATTCAGTTGGTTTGGATTTATTGCCGGTC
**CDR1 IL3 A1271G/R**	GACCGGCAATAAATCCAAACCAACTGAATG
**CDR1 IL3 F1272A/F**	CAGTTGGTTTGCAGCTATTGCCGGTC

**CDR1 IL3 F1272A/R**	GACCGGCAATAGCTGCAAACCAACTG
**CDR1 IL3 I1273A/F**	GTTGGTTTGCATTTGCTGCCGGTCAAATTAC
**CDR1 IL3 I1273A/R**	GTAATTTGACCGGCAGCAAATGCAAACCAAC
**CDR1 IL3 A1274G/F**	GGTTTGCATTTATTGGCGGTCAAATTAC
**CDR1 IL3 A1274G/R**	GTAATTTGACCGCCAATAAATGCAAACC
**CDR1 IL3 G1275A/F**	CATTTATTGCCGCTCAAATTACATCAG
**CDR1 IL3 G1275A/R**	CTGATGTAATTTGAGCGGCAATAAATG
**CDR1 IL3 Q1276A/F**	CATTTATTGCCGGTGCAATTACATCAG
**CDR1 IL3 Q1276A/R**	CTGATGTAATTGCACCGGCAATAAATG
**CDR1 IL3 I1277A/F**	GCCGGTCAAGCTACATCAGAAATTC
**CDR1 IL3 I1277A/R**	GAATTTCTGATGTAGCTTGACCGGC
**CDR1 IL3 T1278A/F**	GCCGGTCAAATTGCATCAGAAATTCC
**CDR1 IL3 T1278A/R**	GGAATTTCTGATGCAATTTGACCGGC
**CDR1 IL3 S1279A/F**	CGGTCAAATTACAGCAGAAATTCC
**CDR1 IL3 S1279A/R**	GGAATTTCTGCTGTAATTTGACCG
**Primers for alanine scanning mutagenesis of ICL 4 of CDR1 (9 Residues) (5'-3')**	
**CDR1 M1337A/F**	GGGTCAATTATGTGCGTCTTTCAGTGAATTAG
**CDR1 M1337A/R**	CTAATTCACTGAAAGACGCACATAATTGACCC
**CDR1 S1338A/F**	GGGTCAATTATGTATGGCTTTCAGTGAATTAG
**CDR1 S1338A/R**	CTAATTCACTGAAAGCCATACATAATTGACCC
**CDR1 F1339A/F**	CAATTATGTATGTCTGCCAGTGAATTAGC
**CDR1 F1339A/R**	GCTAATTCACTGGCAGACATACATAATTG
**CDR1 S1340A/F**	GTATGTCTTTCGCTGAATTAGCTGATAATG
**CDR1 S1340A/R**	CATTATCAGCTAATTCAGCGAAAGACATAC
**CDR1 E1341A/F**	GTATGTCTTTCAGTGCATTAGCTGATAATG
**CDR1 E1341A/R**	CATTATCAGCTAATGCACTGAAAGACATAC
**CDR1 L1342A/F**	GTCTTTCAGTGAAGCAGCTGATAATG
**CDR1 L1342A/R**	CATTATCAGCTGCTTCACTGAAAGAC
**CDR1 A1343G/F**	CTTTCAGTGAATTAGGTGATAATGCTGCC
**CDR1 A1343G/R**	GGCAGCATTATCACCTAATTCACTGAAAG
**CDR1 D1344A/F**	CAGTGAATTAGCTGCTAATGCTGCCAATTTG
**CDR1 D1344A/R**	CAAATTGGCAGCATTAGCAGCTAATTCACTG
**CDR1 N1345A/F**	GTGAATTAGCTGATGCTGCTGCCAATTTGGC
**CDR1 N1345A/R**	GCCAAATTGGCAGCAGCATCAGCTAATTCAC
**Other primers used (5'-3')**	
**CDR1 G190R/F**	GTTTTGGGGAGACCCCGTGCTGGTTGTTCCAC
**CDR1 G190R/R**	GTGGAACAACCAGCACGGGGTCTCCCCAAAAC
**CDR1 G187A/F**	CTTACTGTTGTTTTGGCGAGACCCGGTGCTG
**CDR1 G187A/R**	CAGCACCGGGTCTCGCCAAAACAACAGTAAG
**CDR1 P189A/F**	GTTTTGGGGAGAGCCGGTGCTGGTTGTTC
**CDR1 P189A/R**	GAACAACCAGCACCGGCTCTCCCCAAAAC
**CDR1 G192A/F**	GAGACCCGGTGCTGCTTGTTCCACATTG
**CDR1 G192A/R**	CAATGTGGAACAAGCAGCACCGGGTCTC
**CDR1 F933A/F**	GCCTTAGATTCTTCAGCCCAAAGATCAATTGG
**CDR1 F933A/R**	CCAATTGATCTTTGGGCTGAAGAATCTAAGGC
**CDR1 Q942A/F**	GGTTATGTCCAAGCACAAGATGTTCATTTAC
**CDR1 Q942A/R**	GTAAATGAACATCTTGTGCTTGGACATAACC
**CDR1 D944A/F**	GTCCAACAACAAGCTGTTCATTTACCTAC
**CDR1 D944A/R**	GTAGGTAAATGAACAGCTTGTTGTTGGAC
**CDR1 H946A/F**	CAACAACAAGATGTTGCTTTACCTACATCTAC
**CDR1 H946A/R**	GTAGATGTAGGTAAAGCAACATCTTGTTGTTG

Supplementary Table S5: List of strains used in the study:

Strain	Description	Reference
AD1-8u-	MATa pdr1-3hisG ura3 Δyor1::hisG Δsnq2::hisG Δpdr10::hisG Δpdr11::hisG Δycf1::hisG Δpdr15::hisG	1
CaCdr1p	AD1-8u- cells harbouring CDR1-GFP ORF integrated at PDR5 locus	2
S 567A CDR1-GFP	CDR1-GFP harbouring respective mutation in CDR1 ORF and integrated at PDR5 locus	This study
L 568A CDR1-GFP	CDR1-GFP harbouring respective mutation in CDR1 ORF and integrated at PDR5 locus	This study
F 569A CDR1-GFP	CDR1-GFP harbouring respective mutation in CDR1 ORF and integrated at PDR5 locus	This study
E 570A CDR1-GFP	CDR1-GFP harbouring respective mutation in CDR1 ORF and integrated at PDR5 locus	This study
A571G CDR1-GFP	CDR1-GFP harbouring respective mutation in CDR1 ORF and integrated at PDR5 locus	This study
R572A CDR1-GFP	CDR1-GFP harbouring respective mutation in CDR1 ORF and integrated at PDR5 locus	This study
P 573A CDR1-GFP	CDR1-GFP harbouring respective mutation in CDR1 ORF and integrated at PDR5 locus	This study
I 574 A CDR1-GFP	CDR1-GFP harbouring respective mutation in CDR1 ORF and integrated at PDR5 locus	This study
V575A CDR1-GFP	CDR1-GFP harbouring respective mutation in CDR1 ORF and integrated at PDR5 locus	This study
E 576A CDR1-GFP	CDR1-GFP harbouring respective mutation in CDR1 ORF and integrated at PDR5 locus	This study
K577A CDR1-GFP	CDR1-GFP harbouring respective mutation in CDR1 ORF and integrated at PDR5 locus	This study
H578A CDR1-GFP	CDR1-GFP harbouring respective mutation in CDR1 ORF and integrated at PDR5 locus	This study
K579A CDR1-GFP	CDR1-GFP harbouring respective mutation in CDR1 ORF and integrated at PDR5 locus	This study
K580A CDR1-GFP	CDR1-GFP harbouring respective mutation in CDR1 ORF and integrated at PDR5 locus	This study
Y581A CDR1-GFP	CDR1-GFP harbouring respective mutation in CDR1 ORF and integrated at PDR5 locus	This study
A582G CDR1-GFP	CDR1-GFP harbouring respective mutation in CDR1 ORF and integrated at PDR5 locus	This study
L583A CDR1-GFP	CDR1-GFP harbouring respective mutation in CDR1 ORF and integrated at PDR5 locus	This study
Y584A CDR1-GFP	CDR1-GFP harbouring respective mutation in CDR1 ORF and integrated at PDR5 locus	This study
R585A CDR1-GFP	CDR1-GFP harbouring respective mutation in CDR1 ORF and integrated at PDR5 locus	This study
P 586A CDR1-GFP	CDR1-GFP harbouring respective mutation in CDR1 ORF and integrated at PDR5 locus	This study
S 587A CDR1-GFP	CDR1-GFP harbouring respective mutation in CDR1 ORF and integrated at PDR5 locus	This study

A588G CDR1-GFP	CDR1-GFP harbouring respective mutation in CDR1 ORF and integrated at PDR5 locus	This study
D589A CDR1-GFP	CDR1-GFP harbouring respective mutation in CDR1 ORF and integrated at PDR5 locus	This study
A590G CDR1-GFP	CDR1-GFP harbouring respective mutation in CDR1 ORF and integrated at PDR5 locus	This study
L591A CDR1-GFP	CDR1-GFP harbouring respective mutation in CDR1 ORF and integrated at PDR5 locus	This study
A592G CDR1-GFP	CDR1-GFP harbouring respective mutation in CDR1 ORF and integrated at PDR5 locus	This study
S593A CDR1-GFP	CDR1-GFP harbouring respective mutation in CDR1 ORF and integrated at PDR5 locus	This study
I 594A CDR1-GFP	CDR1-GFP harbouring respective mutation in CDR1 ORF and integrated at PDR5 locus	This study
I 595A CDR1-GFP	CDR1-GFP harbouring respective mutation in CDR1 ORF and integrated at PDR5 locus	This study
S 596A CDR1-GFP	CDR1-GFP harbouring respective mutation in CDR1 ORF and integrated at PDR5 locus	This study
E 597A CDR1-GFP	CDR1-GFP harbouring respective mutation in CDR1 ORF and integrated at PDR5 locus	This study
S645A CDR1-GFP	CDR1-GFP harbouring respective mutation in CDR1 ORF and integrated at PDR5 locus	This study
I646A CDR1-GFP	CDR1-GFP harbouring respective mutation in CDR1 ORF and integrated at PDR5 locus	This study
G647A CDR1-GFP	CDR1-GFP harbouring respective mutation in CDR1 ORF and integrated at PDR5 locus	This study
A648G CDR1-GFP	CDR1-GFP harbouring respective mutation in CDR1 ORF and integrated at PDR5 locus	This study
V649A CDR1-GFP	CDR1-GFP harbouring respective mutation in CDR1 ORF and integrated at PDR5 locus	This study
S650A CDR1-GFP	CDR1-GFP harbouring respective mutation in CDR1 ORF and integrated at PDR5 locus	This study
T651A CDR1-GFP	CDR1-GFP harbouring respective mutation in CDR1 ORF and integrated at PDR5 locus	This study
S652A CDR1-GFP	CDR1-GFP harbouring respective mutation in CDR1 ORF and integrated at PDR5 locus	This study
I653A CDR1-GFP	CDR1-GFP harbouring respective mutation in CDR1 ORF and integrated at PDR5 locus	This study
S654A CDR1-GFP	CDR1-GFP harbouring respective mutation in CDR1 ORF and integrated at PDR5 locus	This study
G655A CDR1-GFP	CDR1-GFP harbouring respective mutation in CDR1 ORF and integrated at PDR5 locus	This study
A656G CDR1-GFP	CDR1-GFP harbouring respective mutation in CDR1 ORF and integrated at PDR5 locus	This study
M657ACDR1-GFP	CDR1-GFP harbouring respective mutation in CDR1 ORF and integrated at PDR5 locus	This study
1248ACDR1-GFP P	CDR1-GFP harbouring respective mutation in CDR1 ORF and integrated at PDR5 locus	This study
Y1249ACDR1-GFP	CDR1-GFP harbouring respective mutation in CDR1 ORF and integrated at PDR5 locus	This study
F1250ACDR1-GFP	CDR1-GFP harbouring respective mutation in CDR1 ORF and integrated at PDR5 locus	This study

V1251ACDR1-GFP	CDR1-GFP harbouring respective mutation in CDR1 ORF and integrated at PDR5 locus	This study
K1252ACDR1-GFP	CDR1-GFP harbouring respective mutation in CDR1 ORF and integrated at PDR5 locus	This study
Q1253ACDR1-GFP	CDR1-GFP harbouring respective mutation in CDR1 ORF and integrated at PDR5 locus	This study
R1254ACDR1-GFP	CDR1-GFP harbouring respective mutation in CDR1 ORF and integrated at PDR5 locus	This study
D1255ACDR1-GFP	CDR1-GFP harbouring respective mutation in CDR1 ORF and integrated at PDR5 locus	This study
V1256ACDR1-GFP	CDR1-GFP harbouring respective mutation in CDR1 ORF and integrated at PDR5 locus	This study
Y1257ACDR1-GFP	CDR1-GFP harbouring respective mutation in CDR1 ORF and integrated at PDR5 locus	This study
E1258A CDR1-GFP	CDR1-GFP harbouring respective mutation in CDR1 ORF and integrated at PDR5 locus	This study
V1259ACDR1-GFP	CDR1-GFP harbouring respective mutation in CDR1 ORF and integrated at PDR5 locus	This study
R1260ACDR1-GFP	CDR1-GFP harbouring respective mutation in CDR1 ORF and integrated at PDR5 locus	This study
E1261ACDR1-GFP	CDR1-GFP harbouring respective mutation in CDR1 ORF and integrated at PDR5 locus	This study
A1262GCDR1-GFP	CDR1-GFP harbouring respective mutation in CDR1 ORF and integrated at PDR5 locus	This study
P1263ACDR1-GFP	CDR1-GFP harbouring respective mutation in CDR1 ORF and integrated at PDR5 locus	This study
S1264ACDR1-GFP	CDR1-GFP harbouring respective mutation in CDR1 ORF and integrated at PDR5 locus	This study
R1265ACDR1-GFP	CDR1-GFP harbouring respective mutation in CDR1 ORF and integrated at PDR5 locus	This study
T1266ACDR1-GFP	CDR1-GFP harbouring respective mutation in CDR1 ORF and integrated at PDR5 locus	This study
F1267ACDR1-GFP	CDR1-GFP harbouring respective mutation in CDR1 ORF and integrated at PDR5 locus	This study
S1268ACDR1-GFP	CDR1-GFP harbouring respective mutation in CDR1 ORF and integrated at PDR5 locus	This study
W1269ACDR1-GFP	CDR1-GFP harbouring respective mutation in CDR1 ORF and integrated at PDR5 locus	This study
F1270ACDR1-GFP	CDR1-GFP harbouring respective mutation in CDR1 ORF and integrated at PDR5 locus	This study
A1271GCDR1-GFP	CDR1-GFP harbouring respective mutation in CDR1 ORF and integrated at PDR5 locus	This study
A1271GCDR1-GFP	CDR1-GFP harbouring respective mutation in CDR1 ORF and integrated at PDR5 locus	This study
F1272ACDR1-GFP	CDR1-GFP harbouring respective mutation in CDR1 ORF and integrated at PDR5 locus	This study
I1273ACDR1-GFP	CDR1-GFP harbouring respective mutation in CDR1 ORF and integrated at PDR5 locus	This study
A1274GCDR1-GFP	CDR1-GFP harbouring respective mutation in CDR1 ORF and integrated at PDR5 locus	This study
G1275ACDR1-GFP	CDR1-GFP harbouring respective mutation in CDR1 ORF and integrated at PDR5 locus	This study

Q1276ACDR1-GFP	CDR1-GFP harbouring respective mutation in CDR1 ORF and integrated at PDR5 locus	This study
I1277ACDR1-GFP	CDR1-GFP harbouring respective mutation in CDR1 ORF and integrated at PDR5 locus	This study
T1278ACDR1-GFP	CDR1-GFP harbouring respective mutation in CDR1 ORF and integrated at PDR5 locus	This study
S1279ACDR1-GFP	CDR1-GFP harbouring respective mutation in CDR1 ORF and integrated at PDR5 locus	This study
M1337ACDR1-GFP	CDR1-GFP harbouring respective mutation in CDR1 ORF and integrated at PDR5 locus	This study
S 1338ACDR1-GFP	CDR1-GFP harbouring respective mutation in CDR1 ORF and integrated at PDR5 locus	This study
F 1339ACDR1-GFP	CDR1-GFP harbouring respective mutation in CDR1 ORF and integrated at PDR5 locus	This study
S1340ACDR1-GFP	CDR1-GFP harbouring respective mutation in CDR1 ORF and integrated at PDR5 locus	This study
E1341ACDR1-GFP	CDR1-GFP harbouring respective mutation in CDR1 ORF and integrated at PDR5 locus	This study
L1342ACDR1-GFP	CDR1-GFP harbouring respective mutation in CDR1 ORF and integrated at PDR5 locus	This study
A1343GCDR1-GFP	CDR1-GFP harbouring respective mutation in CDR1 ORF and integrated at PDR5 locus	This study
D1344ACDR1-GFP	CDR1-GFP harbouring respective mutation in CDR1 ORF and integrated at PDR5 locus	This study
N1345ACDR1-GFP	CDR1-GFP harbouring respective mutation in CDR1 ORF and integrated at PDR5 locus	This study
G190R CDR1-GFP	CDR1-GFP harbouring respective mutation in CDR1 ORF and integrated at PDR5 locus	This study
G190R CDR1-GFP	CDR1-GFP harbouring respective mutation in CDR1 ORF and integrated at PDR5 locus	This study
G187A CDR1-GFP	CDR1-GFP harbouring respective mutation in CDR1 ORF and integrated at PDR5 locus	This study
G187A CDR1-GFP	CDR1-GFP harbouring respective mutation in CDR1 ORF and integrated at PDR5 locus	This study
P189A CDR1-GFP	CDR1-GFP harbouring respective mutation in CDR1 ORF and integrated at PDR5 locus	This study
P189A CDR1-GFP	CDR1-GFP harbouring respective mutation in CDR1 ORF and integrated at PDR5 locus	This study
G192A CDR1-GFP	CDR1-GFP harbouring respective mutation in CDR1 ORF and integrated at PDR5 locus	This study
G192A CDR1-GFP	CDR1-GFP harbouring respective mutation in CDR1 ORF and integrated at PDR5 locus	This study
F933A CDR1-GFP	CDR1-GFP harbouring respective mutation in CDR1 ORF and integrated at PDR5 locus	This study
F933A CDR1-GFP	CDR1-GFP harbouring respective mutation in CDR1 ORF and integrated at PDR5 locus	This study
Q942A CDR1-GFP	CDR1-GFP harbouring respective mutation in CDR1 ORF and integrated at PDR5 locus	This study
Q942A CDR1-GFP	CDR1-GFP harbouring respective mutation in CDR1 ORF and integrated at PDR5 locus	This study
D944A CDR1-GFP	CDR1-GFP harbouring respective mutation in CDR1 ORF and integrated at PDR5 locus	This study

D944A CDR1-GFP	CDR1-GFP harbouring respective mutation in CDR1 ORF and integrated at PDR5 locus	This study
H946A CDR1-GFP	CDR1-GFP harbouring respective mutation in CDR1 ORF and integrated at PDR5 locus	This study
H946A CDR1-GFP	CDR1-GFP harbouring respective mutation in CDR1 ORF and integrated at PDR5 locus	This study

Supplementary Figure S1: Phenotype of 85 ICL alanine/glycine mutants of Cdr1p as seen in drug susceptibility Spot assay. A) Spot assay for ICL1 mutants of CDR1-GFP B) Spot assay for ICL2 mutants of CDR1-GFP C) Spot assay for ICL3 mutants of CDR1-GFP D) Spot assay for ICL4 mutants of CDR1-GFP. Drug susceptibility was checked in presence of 8 drugs at indicated concentrations.

A)

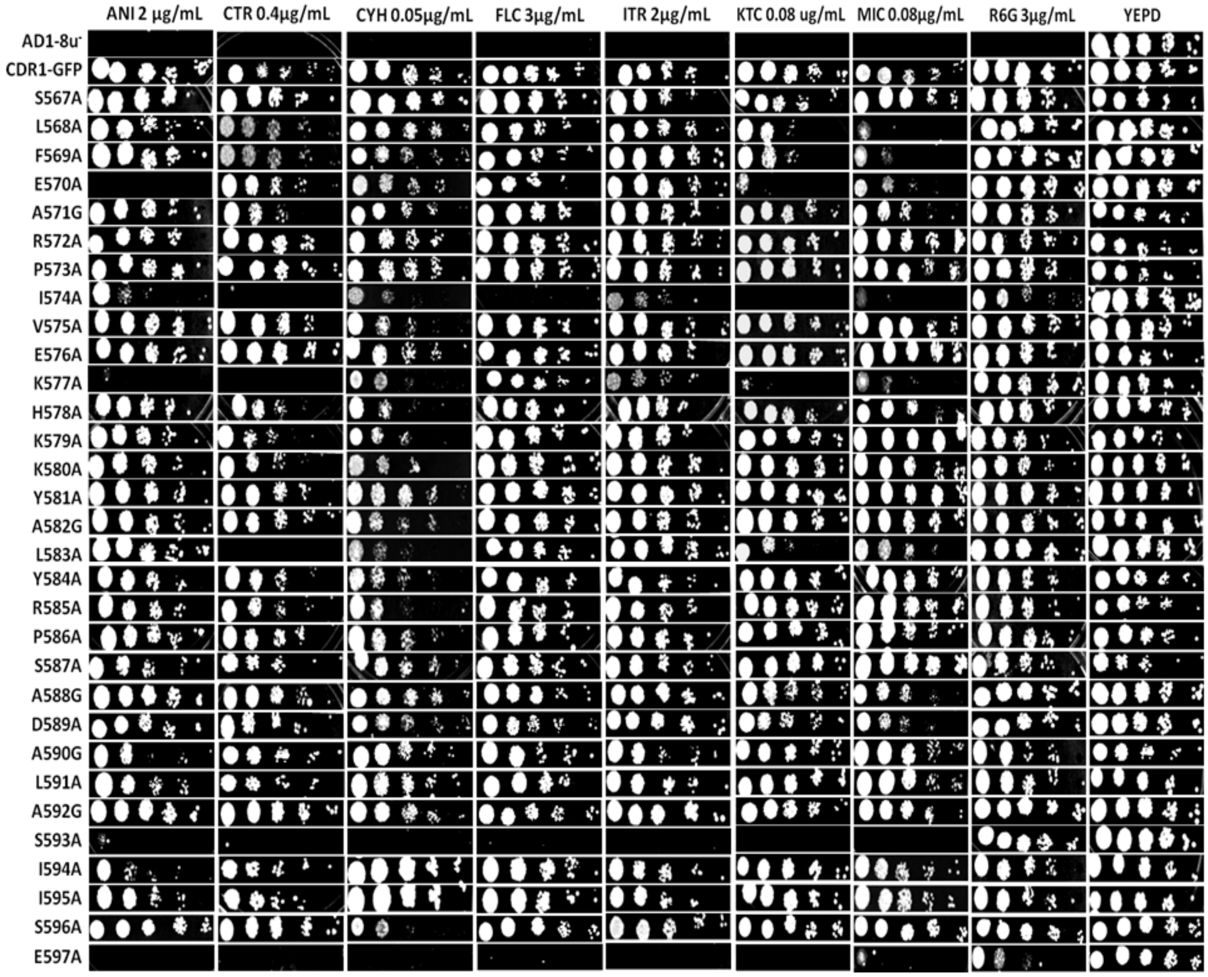

B)

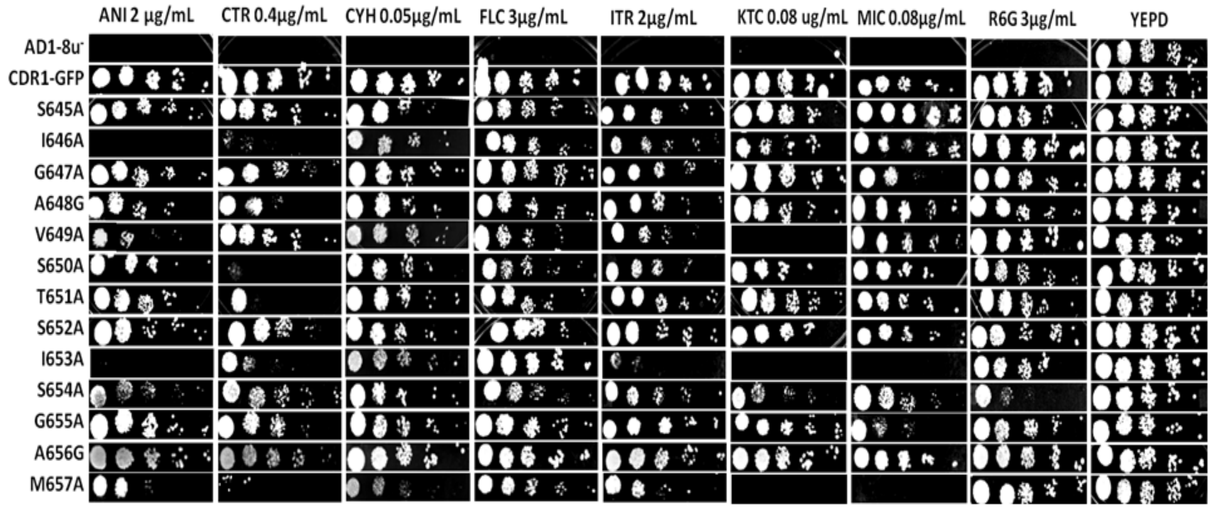
ANI 2 µg/mL
CTR 0.4µg/mL
CYH 0.05µg/mL
FLC 3µg/mL
ITR 2µg/mL
KTC 0.08 ug/mL
MIC 0.08µg/mL
R6G 3µg/mL
YEPD
AD1-8u-
CDR1-GFP
S645A
I646A
G647A
A648G
V649A
S650A
T651A
S652A
I653A
S654A
G655A
A656G
M657A

C)

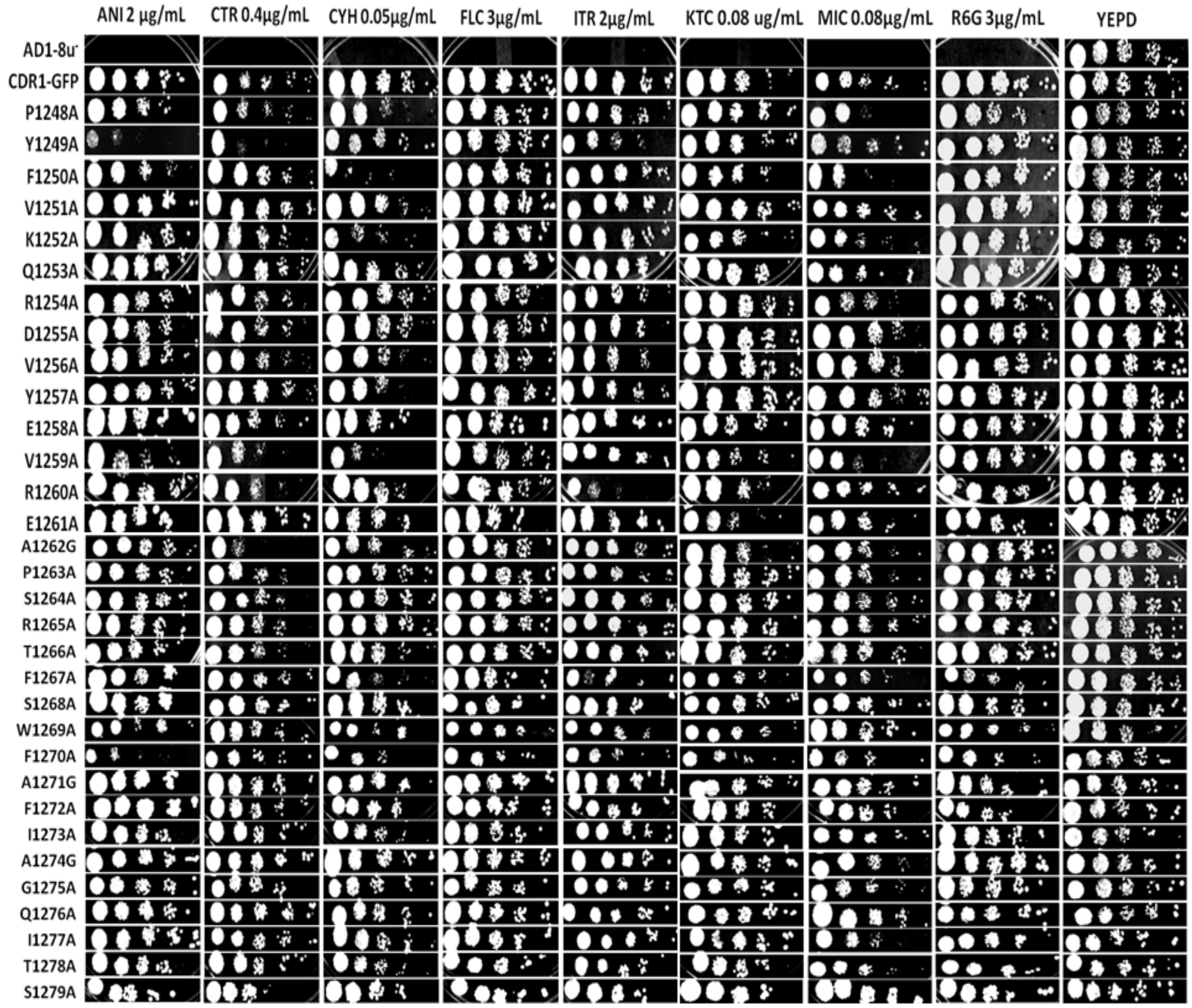
ANI 2 µg/mL
CTR 0.4µg/mL
CYH 0.05µg/mL
FLC 3µg/mL
ITR 2µg/mL
KTC 0.08 ug/mL
MIC 0.08µg/mL
R6G 3µg/mL
YEPD
AD1-8u-
CDR1-GFP
P1248A
Y1249A
F1250A
V1251A
K1252A
Q1253A
R1254A
D1255A
V1256A
Y1257A
E1258A
V1259A
R1260A
E1261A
A1262G
P1263A
S1264A
R1265A
T1266A
F1267A
S1268A
W1269A
F1270A
A1271G
F1272A
I1273A
A1274G
G1275A
Q1276A
I1277A
T1278A
S1279A

D)

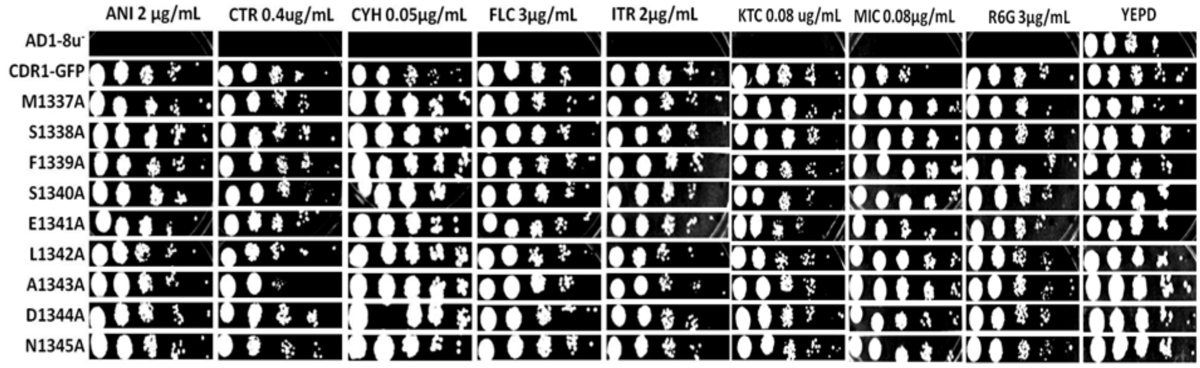

Supplementary Figure S2: Trypsinization of drug susceptible alanine mutants of ICLs. Limited proteolysis of highly drug susceptible mutants of Cdr1p. Limited trypsinization of the 40 μg protein from PM fractions containing Cdr1p-GFP or its ICL mutant variants was performed at 4°C in 50 mM Tris-Cl buffer, pH 7.5 as described in Experimental Procedures. Digestion pattern was obtained by immunoblotting against GFP tagged protein.

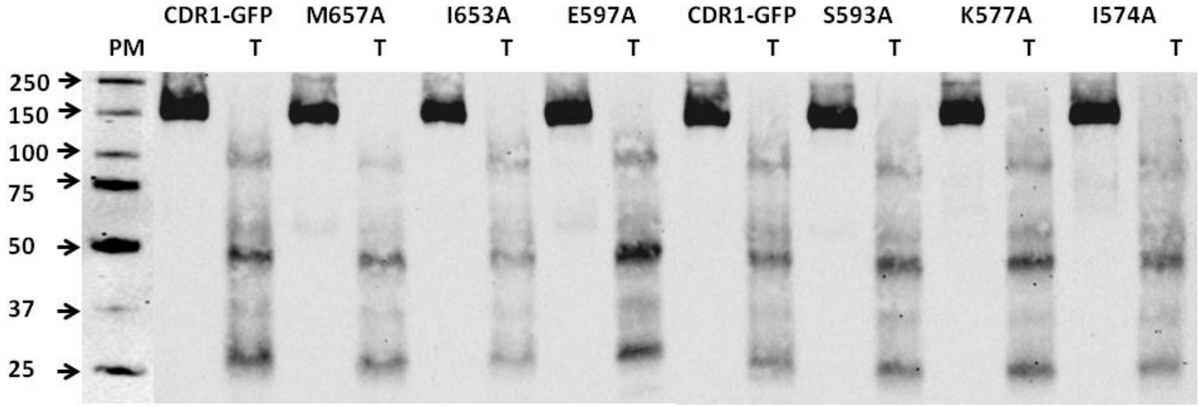

References

1. Decottignies, A. *et al*. ATPase and multidrug transport activities of the overexpressed yeast ABC protein Yor1p. *J Biol Chem.* **273**,12612-22 (1998).
2. Shukla, S. *et al*. Functional characterization of *Candida albicans* ABC transporter Cdr1p. *Eukaryot Cell*. **2**,1361-75 (2003).
